# Housing and displacement as risk factors for negative health outcomes among people who inject drugs in Los Angeles, CA, and Denver, CO, USA

**DOI:** 10.1186/s12889-026-26200-2

**Published:** 2026-01-31

**Authors:** Jesse Lloyd Goldshear, Karen F. Corsi, Rachel Carmen Ceasar, Siddhi S. Ganesh, Kelsey A. Simpson, Alex H. Kral, Ricky N. Bluthenthal

**Affiliations:** 1 Department of Population and Public Health Sciences, Keck School of Medicine, University of Southern California, 1845 N. Soto Street, Los Angeles, CA 90032 USA; 2 Division of Infectious Diseases & Global Public Health, Department of Medicine, University of California San Diego School of Medicine, 9500 Gilman Drive, La Jolla, CA 92093 USA; 3 Department of Psychiatry, School of Medicine, University of Colorado, 1890 N. Revere Court, Suite 5242, Aurora, CO 90045 USA; 4 RTI International, 2150 Shattuck Avenue, Suite 800, Berkeley, CA 94704 USA

**Keywords:** Homelessness, Forced displacement, People who inject drugs, Drug overdose, Risk behaviors, Homelessness policy

## Abstract

**Background:**

The United States is currently experiencing a housing and homelessness crisis. In response, many cities have adopted policies of displacement that move unhoused people from place to place. Recent research indicates that these policies may have negative health impacts on unhoused people who use drugs. We sought to examine health risks associated with forced displacement among unhoused people who inject drugs (PWID).

**Methods:**

We interviewed a community-recruited sample of opioid-using PWID in Los Angeles, CA and Denver, CO between April 2021 and November 2022 (*N* = 472) about their demographic/socioeconomic characteristics, drug use patterns, housing status, forced displacement including items discarded during displacements, and health risks. We constructed binomial generalized linear regression to examine the risk ratio of non-fatal overdose, and syringe and cooker/cotton sharing between four groups of participants: housed, unhoused and not displaced, unhoused and moved, and unhoused and displaced in the last three months.

**Results:**

In the last 3 months, 52% of participants were unhoused and displaced by the government. Among those who were displaced, median number of forced displacements was 3 with 69% reporting loss of syringes, 56% loss of naloxone, and 22% loss of buprenorphine medicine. In multivariable models, adjusted risk ratios for unhoused and displaced participants were higher for nonfatal overdose (ARR = 3.84, 95% CI: 1.60–9.20), cooker/cotton sharing (ARR = 2.46, 95% CI: 1.40–4.33), and syringe sharing (ARR = 2.76, 95% CI: 1.04–7.29) as compared to housed participants.

**Conclusions:**

Unhoused and displaced PWID experience elevated health risks. Ending the use of forced displacement of unhoused PWID is essential to reducing health risk in this population.

**Supplementary Information:**

The online version contains supplementary material available at 10.1186/s12889-026-26200-2.

## Introduction

North America is currently in the midst of two intersecting crises – increasing houselessness and increasing opioid and polysubstance involved overdose deaths [1]. The causes of these twin crises are multifaceted, including the rising cost of housing and growing financial inequality [2], rising medical costs and other debt [3], the increasing potency and availability of synthetic opioids like fentanyl [4], and an increasingly volatile, and unregulated drug supply [5]. In 2022, over 100,000 overdose deaths were reported in the United States [6]. In 2023 the federal Department of Housing and Urban Development (HUD) reported a single-day estimate of 653,104 currently unhoused individuals across the U.S [7]. Among this estimate includes a growing population of people who are *unsheltered*, a status defined by HUD as “residing in a place unfit for human habitation” which includes vehicles, abandoned buildings, and in public areas such as sidewalks and parks [8]. HUD estimated that in 2023, 108,035 (16.5%) unhoused people struggled with “chronic” drug use [9]. 

Among unhoused people who use drugs, health risks are often elevated due to a lack of material resources, difficulty accessing hygiene, environmental exposure, and general social abandonment. These health risks include a heightened risk of overdose – fatal and nonfatal – and an increased likelihood of bloodborne infections and skin and soft tissue infections [10]. While many urban and nonurban municipalities have attempted to address these health risks by adopting harm reduction policies [11], structural interventions to address the growing houselessness contributing to these risk factors appear to be less available [12]. 

Instead, policies of forced displacement and ‘urban purification’ have become widely recognized as viable strategies for removing unhoused people from public sight [13]. Commonly referred to as *sweeps*, these policies are not new. In cities like Los Angeles, one of the locations of this study, so-called anti-camping and similar ordinances have been utilized for decades to move unhoused individuals from busier and more affluent neighborhoods to Skid Row, an area of concentrated poverty and houselessness in downtown Los Angeles, among other locations [14]. Often conducted with minimal notice, unhoused community members are forced to rapidly pack up and move their belongings or risk having most of their possessions discarded or confiscated. This is usually done under the threat of arrest. While the use of these government enforced sweeps by Los Angeles City was under temporary moratorium during the early stages of the COVID-19 pandemic, their deployment re-emerged in 2021. Other municipalities such as Denver, Colorado, the other location for this study, continued to use forced displacement throughout the pandemic. Given the recent US Supreme Court case *City of Grants Pass v. Johnson*—in which justices ruled that polices of displacement were constitutional even when cities do not provide other shelter alternatives—policies of displacement are likely to be even more widely adopted [15]. 

Beyond the displacement of large encampment communities, anti-camping and public safety ordinances are also used to displace more isolated individuals and those living in other types of outdoor shelter including vehicles. These smaller scale displacements are likely no less harmful, as loss of personal property and relocation to an unfamiliar area can still occur. As with larger displacement events, meaningful connection to social services is rare. Vehicle occupants are often ticketed, and fines amass until the vehicle is impounded along with any belongings left inside. Their owners are often unable to afford the fees to retrieve them, losing both their shelter and belongings [16]. While local police (or, in Los Angeles, also sheriffs) are often the agents responsible for enforcing these displacement policies, private security guards have become increasingly involved in surveilling and removing unhoused people from public and “mass private” spaces [17, 18]. As they lack access to truly private spaces, unhoused people often make use of, and reside in, these quasi-public spaces patrolled by security personnel. At the behest of businesses in these spaces, security personnel may expand their scope into more public areas [19]. In some of these hybrid private-public locations security guards become responsible for driving away unhoused people who set up shelters either by force, or by calling for an official displacement [20]. In this way they serve as extensions of law enforcement.

When provided notice, unhoused people who use drugs may also move from an established location before displacement occurs to avoid losing their possessions or being subject to violence. Unhoused people may also choose to relocate periodically for a variety of reasons including hostility from housed neighbors, other changes in the local environment, or wishing to avoid contact with law enforcement [21]. We define these moves as *self-directed*, in that while these moves occur without the immediate threat of violence, they may still be coerced or influenced by other social, material, and political environmental characteristics. These moves still represent a source of disruption and may still include loss of property and disruption of community networks.

For people who use drugs, forced displacement may represent a distinct source of health risk [22, 23]. A recent simulation study indicated that policies of continual forced displacement could contribute to thousands of additional deaths among this population, as well as reduce the initiation of medication assisted treatment [24]. Additional recent ethnographic work by members of this team has shown the negative psychological impact of forced displacement and confiscation of belongings [21]. In this analysis, we examined if experiences of displacement (forced and self-directed) were associated with health outcomes (non-fatal overdose) and risk behaviors (sharing syringes and cooker/cotton) among people who inject drugs (PWID) in Los Angeles and Denver.

## Methods

### Sampling and recruitment

Between April 2021 and November 2022 people who inject drugs (PWID) were recruited from community settings and service organizations that are commonly used by PWID including syringe service sites, public shower, bathroom, and laundry facilities for unhoused people, and other community locations frequented by PWID in Los Angeles, CA and Denver, CO. Study eligibility included being 18 years of age or older, self-reported opioid use in the last 30 days, and self-reported drug injection within the past 30 days, which was confirmed by visual inspection of injection sites. After providing informed consent, participants answered questions on demographic and socio-economic characteristics (including unstable housing and displacement), drug use patterns, drug treatment involvement, and other items in a one-on-one computer assisted personal interview using the Questionnaire Development System (Nova Research, Bethesda, MD). Participants received $20 for completing this interview. This cross-sectional analysis includes data from 472 PWID, of which 223 from Los Angeles and 249 from Denver. All study procedures were reviewed and approved by the institutional review board at the University of Southern California.

### Study measures

The study questionnaire was developed for this study (Supplemental File 1), and includes modified measures from the graded chronic pain scale [25], the National Institutes on Drug Abuse risk behavior assessment [26, 27], the Pittsburgh sleep quality index [28], and questions about subsistence from Gelberg et al., [29]. Item numbers from the questionnaire are noted throughout for ease of reference.

### Exposure variables

We were interested in how forced displacement impacted health outcomes and risk among PWID. We created a categorical variable representing both housing status and displacement type. For participants who reported being unhoused, spending at least one night in the last three months in a tent, outdoors, vehicle, abandon building/garage/shed, (Item A16) and who reported at least one move in that time (Item A17), we asked, “In the last 3 months, how many of these moves were because of police, security guards, or other city/county officials?” (Item A18) Participants reporting one or more government-involved moves were classified as “unhoused and displaced by the government.” Participants reporting at least one move but zero due to the government were classified as “unhoused and moved”. Those reporting being unhoused and not moving for any reason were classified as “unhoused and not moved.” The remaining participants were classified as “housed.”

Participants were also asked if any items had been discarded by “city, state, or county employees such as police or sanitation workers?” in the past three months (Items A19 and A20). The item listed included identification, other important paperwork, Naloxone/Narcan, Suboxone/Subutex (buprenorphine), chronic condition medication (e.g., diabetes), acute condition medication (e.g. bacterial infection), clothing, food, illicit drugs, photos, pets, syringes, cotton or cookers (paraphernalia), biohazard containers, and tents or shelter. This list included material that is important for reducing fatal overdose risk, HIV and HCV transmission risk, and for maintaining opioid treatment and withdrawal management.

### Outcome variables

Outcome variables for this analysis included nonfatal overdose, receptive syringe sharing, and sharing cotton or cookers. Nonfatal overdose was assessed as an affirmative answer to: “In the last 3 months, have you overdosed?” (Item D1) Participants who reported one or more instances of injecting ”…using syringes/needles that you know had been used by someone else (including a close friend or lover)?” were classified as having shared syringes (Item C6). Participants who responded yes to having “…shared a cooker, spoon, or shaker that someone else used before you?” (Item C7) and/or “…have used a filter or cotton that someone else used before you?” (Item C9) were classified as having shared a cooker or cotton.

### Potentially confounding variables

We considered the following demographic and socioeconomic variables as potential covariates in this study: gender (male or not male, [Item A3]), race/ethnicity (White, Black, Latinx, Native American, Asian or Pacific Islander, Mixed [Items A5 and A6]), age (< 30, 30 to 39, 40 to 49, or 50 or older [Items A1 and A2)), education (less than high school education or more [Item A9]), and monthly income (<$1,000, $1,001 to $1,400, or 1,401 to $2,100, or $2,101 or more [Item A7]). We chose to categorize age in ten-year increments in line with our other publications from this cohort. In-line with previous observational studies from members of this group, we categorized our income item according to federal poverty guidelines at the time of questionnaire development.

Drug use measures included injection frequency, and types and times of drugs used by route (injection or non-injection). We converted injection frequency in the last three months (Calculated from Items B12, B17, B22, B27, B32, B37, B47, B42, B52, B57, B62, B67, and B72) into a categorical variable with the following classifications: less than daily use (< 89 injections), at least once but less than three times a day (90 to 269 injections), and three or more times a day ($\:\ge\:$270 injections). We re-categorized rushed injection frequency (Item C20) as none, 1 to 9 times (weekly), and 10 or more times (more than weekly). We also considered factors that have been associated with health outcome variables including contact with security guards, police and other criminal justice involvement (e.g. parole and probation, Items I1 – I9) [19, 30], receiving assistance with injection (Items C17 and C18), syringe access and syringe reuse (Items C1 and C4) [31, 32], withdrawal symptoms from opioids and methamphetamine (Items E1 and E4) [33, 34], rushed injection (Item C20) [35, 36], enrollment in substance use treatment (e.g. current enrollment and enrollment in the last three months in methadone, buprenorphine, residential, and outpatient treatment, [Items J1 – J4]), and any mental health disorder diagnosis (Item G36).

### Statistical analysis

After descriptive analyses, we conducted bivariate analyses to determine factors associated at the *p* < 0.05 level with nonfatal overdose, receptive syringe sharing, and sharing cookers or cotton using chi-square tests for categorical variables and t-tests for continuous variables. We considered the variables listed above in the domains of demographics, socioeconomic characteristics, displacement, and substance use behaviors as potential correlates and confounders in the relationship between displacement and the above outcome variables. Variables significant in bivariate analyses were then assessed for collinearity within domains using Pearson correlation coefficients. Collinear variables (Pearson correlation coefficient > 0.30) were included in the final analyses as confounders if they were also significantly associated with the displacement exposure variable.

We used stepwise binomial generalized linear regression to calculate adjusted risk ratios (ARR) for the outcome variables while controlling for confounding [37, 38]. Variables not significant in multivariable analyses (*p* > 0.05) at each step were removed from models with the exception of recruitment city. We included recruitment city in all final multivariable models due to statistically significant differences in forced displacement by city (*p* < 0.001). We also used binomial generalized linear regression to calculate unadjusted risk of item confiscation (e.g. naloxone, government ID) by movement category. For all models, ARR/RR and 95% Confidence Intervals (CI) were estimated. In all regression models, a reference category was chosen for comparison (e.g. housed was the reference category for movement status, none was the reference category for rushed injection frequency). All analyses were performed using SPSS, Version 25 [39]. 

## Results

Our sample consisted of 472 participants (249 from Denver and 223 from Los Angeles), of which 77% were male, 52% were White, 26% were Latinx, 10% were Native American, and 6% were Black (Table 1). Participants were most frequently between the ages of 30 and 39 (35%) years old, followed by 25% who were over 50 years old, and 24% who were between 40 and 49 years old. The majority were unstably housed (84%), high school educated (77%), single (72%), and low income (53% had monthly income of less than $1,000 USD). In terms of drug use, 86% reported daily opioid use (including heroin, fentanyl, and/or prescription opioids), 70% reported daily injection, and 59% reported daily methamphetamine use.

**Table 1:** Selected demographic, socioeconomic, drug use and health characteristics of opioid-using people who inject drugs in Denver, CO and Los Angeles, CA, 2021/22 (*N* = 472)

Characteristics	Total *N* = 472 %	Housed *N* = 77 %	Unhoused not moved *n* = 99 %	Unhoused, moved *n* = 51 %	Unhoused, displaced *n* = 245 %
Site*					
Denver	249 (53%)	21 (27%)	34 (34%)	22 (43%)	172 (70%)
Los Angeles	223 (47%)	56 (73%)	65 (66%)	29 (57%)	73 (30%)
Gender					
Male	364 (77%)	52 (68%)	79 (80%)	38 (75%)	195 (80%)
Race*					
White	246 (52%)	34 (44%)	42 (42%)	23 (45%)	147 (60%)
Latinx	123 (26%)	29 (38%)	37 (37%)	16 (31%)	41 (17%)
African American	26 (6%)	5 (7%)	8 (8%)	2 (4%)	11 (4%)
Asian/Pacific Islander	5 (1%)	0 (0%)	0 (0%)	0 (0%)	5 (2%)
Native American	49 (10%)	4 (5%)	4 (4%)	8 (16%)	31 (13%)
Mixed Race	25 (5%)	5 (6%)	8 (8%)	2 (4%)	10 (4%)
Age (years)*					
<30	76 (16%)	7 (9%)	11 (11%)	11 (21%)	47 (19%)
30–39	164 (35%)	16 (21%)	27 (27%)	20 (39%)	101 (41%)
40–49	115 (24%)	20 (26%)	26 (26%)	10 (20%)	59 (24%)
≥50	117 (25%)	34 (44%)	35 (35%)	10 (20%)	38 (16%)
High school education or more	365 (77%)	58 (75%)	71 (72%)	38 (75%)	198 (81%)
Monthly Income					
< $1,000	249 (53%)	36 (47%)	49 (51%)	34 (66%)	130 (53%)
$1,001 - $1,400	82(19%)	18 (23%)	17 (18%)	7 (14%)	45 (18%)
$1,401 - $2,100	67 (13%)	8 (10%)	17 (18%)	4 (8%)	33 (14%)
> $2,101	70 (15%)	15 (20%)	13 (13%)	6 (12%)	36 (15%)
Drug use, last 3 months					
Crack cocaine*	157 (33%)	15 (20%)	25 (25%)	23 (45%)	94 (38%)
Powder cocaine	144 (31%)	17 (22%)	28 (29%)	13 (26%)	86 (35%)
Methamphetamine*	394 (84%)	48 (62%)	81 (82%)	42 (82%)	223 (91%)
Heroin	387 (82%)	68 (88%)	81 (82%)	38 (75%)	200 (82%)
Fentanyl*	329 (70%)	31 (40%)	64 (65%)	35 (69%)	199 (81%)
Speedball*	137 (29%)	16 (21%)	21 (21%)	11 (22%)	89 (36%)
Goofball*	299 (63%)	32 (42%)	57 (58%)	30 (59%)	180 (74%)
Cannabis*	355 (75%)	51 (66%)	69 (70%)	37 (73%)	198 (81%)
Non-prescription use					
Opioids	134 (28%)	17 (22%)	25 (25%)	11 (22%)	81 (33%)
Tranquilizers*	178 (38%)	20 (26%)	31 (31%)	19 (37%)	108 (44%)
Stimulants	59 (13%)	6 (8%)	11 (11%)	5 (10%)	37 (15%)
Methadone	73 (16%)	13 (17%)	8 (8%)	7 (14%)	45 (18%)
Buprenorphine*	64 (14%)	6 (8%)	7 (7%)	7 (14%)	44 (18%)
Rushed injection, last 3 months*					
None	172 (36%)	40 (52%)	39 (39%)	22 (43%)	71 (29%)
1 to 9 times	135 (29%)	21 (27%)	29 (29%)	15 (29%)	70 (29%)
10 times or more	165 (35%)	16 (21%)	31 (31%)	14 (28%)	104 (42%)
Gave an injection to another PWID in the last 3 months*	261 (55%)	19 (25%)	53 (54%)	27 (53%)	162 (66%)
Received an injection from another PWID, last 3 months	158 (34%)	19 (25%)	30 (30%)	14 (28%)	95 (39%)
Any syringe reuse, last 3 months	187 (40%)	23 (30%)	33 (33%)	23 (45%)	108 (44%)
Criminal legal contact, last 3 months					
Police*	206 (44%)	12 (16%)	35 (35%)	19 (37%)	140 (57%)
Parole	23 (5%)	1 (1%)	6 (6%)	3 (6%)	13 (5%)
Probation	48 (10%)	4 (5%)	8 (8%)	4 (8%)	32 (13%)
Arrest*	56 (12%)	0 (0%)	13 (13%)	5 (10%)	38 (16%)
Security guard*	223 (47%)	18 (23%)	41 (42%)	16 (31%)	148 (60%)
Nonfatal overdose, last 3 months*	100 (21%)	5 (7%)	19 (19%)	8 (16%)	68 (28%)
Receptive syringe sharing, last 3 months*	101 (21%)	4 (5%)	18 (18%)	19 (37%)	60 (25%)
Cooker/Cotton sharing, last 3 months*	174 (41%)	11 (14%)	36 (36%)	18 (45%)	109 (49%)

* *p* = < 0.05

In the last three months, 16% (77/472) of participants were housed, 21% (99/472) were unhoused but indicated no displacement, 11% (51/472) were unhoused and moved, 52% (245/472) were unhoused and forcibly displaced. For those people who were unhoused, the mean nights outdoors in the last 3 months was 58.23 (standard deviation [STD] = 33.65; median = 70, interquartile range [IQR] = 25, 90). Among those who moved at least once for any reason in the past three months, the median number of moves was 4 (IQR = 1, 20). The median number of moves due to government displacement was 3 (IQR = 1,10).

**Fig. 1:**
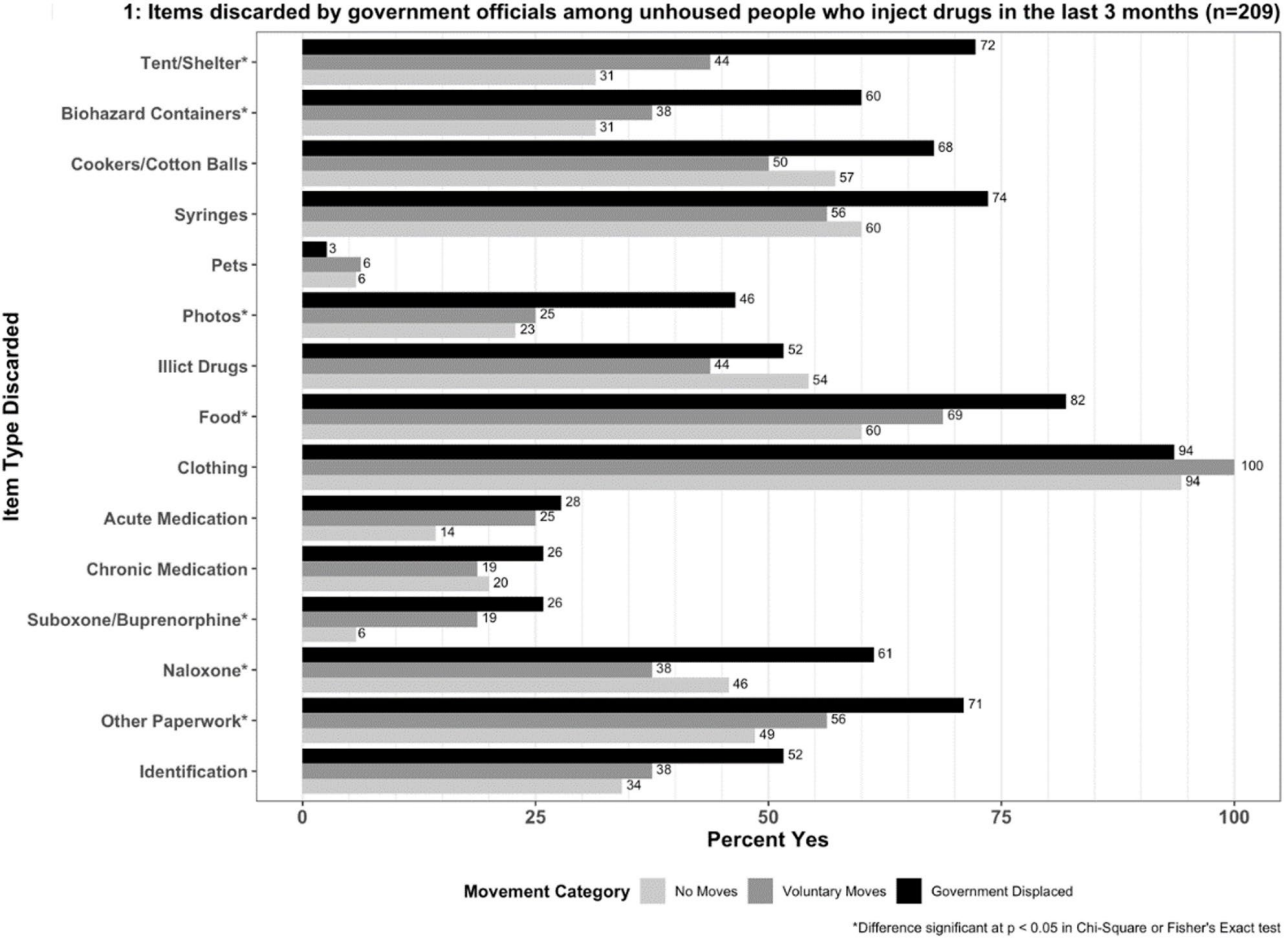
Items discarded by government officials in the last 3 months among unhoused people who inject drugs (*n* = 209). *Differences significant at *p* < 0.05 in Chi-square or Fisher’s Exact tests

Over half (64% or 157/245) of participants who were forcibly displaced reported having items taken by government officials (mean number of items taken 7.56, STD = 3.54 = 2; median = 8; IQR = 5,10) with clothing being the most commonly taken item (93%). Among those who were displaced and had items taken, reports of having health-related items taken at least once in the last three months included syringes (73%) and cookers/cotton (68%) for HIV/HCV prevention, naloxone (60%) for overdose reversal, and buprenorphine (26%) for opioid use disorder (Fig. 1). Participants reporting forced displacement reported higher prevalence of having any items discarded (*p* < 0.05) than participants who were unhoused but not displaced. In chi-square and Fisher exact tests, there were significant differences in prevalence of specific items discarded (paperwork, naloxone, suboxone/buprenorphine, food, photos, biohazard containers, and tents) and displacement category among unhoused individuals. For those items which were significantly different, we produced bivariate models to determine specific risk ratios between item confiscation and displacement category (Table 2). Among unhoused participants who had items taken, those who were subject to government displacement had significantly (*p* < 0.05) increased risk of losing all items listed above except naloxone. We found no statistically significant bivariate associations between reporting having items discarded and any of our three model outcome variables.

**Table 2:** Binomial generalized linear models of displacement category on items discarded in the last 3 months among unhoused people who inject drugs in Denver and Los Angeles (*n* = 209)

Item Taken	No moves (*n* = 99)	Self-directed moves (*n* = 51)	Government Displaced (*n* = 245)
	RR (95% CI)	RR (95% CI)	RR (95% CI)
Paperwork	Referent	1.12 (0.64, 1.97)	1.49 (1.04, 2.14)*
Naloxone	Referent	0.79 (0.38, 1.66)	1.37 (0.93, 2.02)
Suboxone/Buprenorphine	Referent	3.18 (0.58, 17.28)	4.62 (1.17, 18.22)*
Food	Referent	1.11 (0.71, 1.73)	1.40 (1.05, 1.86)*
Photos	Referent	1.06 (0.37, 3.03)	2.08 (1.10, 3.92)*
Biohazard containers	Referent	1.16 (0.51, 2.60)	1.95 (1.17, 3.25)*
Tents	Referent	1.35 (0.64, 2.86)	2.35 (1.42, 3.88)*

After adjusting for recruitment site and other confounding variables, being unhoused and not displaced significantly increased risk of nonfatal overdose (RR = 2.71, 95% Confidence Interval [CI] = 1.07, 6.82, *p* = 0.03), as did being unhoused and displaced by government action (RR = 3.84, 95% CI = 1.60, 9.20, *p* = 0.002) when compared to housed participants (Table 3).

**Table 3:** Binomial generalized linear model of nonfatal overdose in the last 3 months among people who inject drugs in Denver and Los Angeles (*N* = 472)

Factor	Adjusted Risk Ratio	95% confidence interval	*p*-value
Housing status			
Housed	Referent	-	-
Unhoused, not moved	2.70	1.07, 6.82	0.035
Unhoused, self-directed moves	2.52	0.89, 7.14	0.082
Unhoused, forcibly displaced	3.84	1.60, 9.20	0.002
Rushed injection in the last 3 months			
Not rushed	Referent	-	-
Rushed 1 to 9 times	1.81	1.11, 2.99	0.018
Rushed 10 or more times	2.04	1.26, 3.28	0.003
Injected by another person in the last 3 months			
No	Referent	-	-
Yes	1.70	1.21, 2.37	0.002
Recruitment Location			
Denver	Referent	-	-
Los Angeles	1.39	0.99, 1.95	0.058

In a multivariable model that adjusted for confounding variables, including recruitment site (Table 4), being unhoused and self-directedly moving (RR = 4.54, 95% CI = 1.68, 12.27, *p* = 0.003) and being displaced (RR = 2.76, 95% CI = 1.04, 7.29, *p* = 0.041) were significantly associated with increased risk of receptive syringe sharing as compared to housed participants.

**Table 4:** Binomial generalized linear model of receptive syringe sharing in the last 3 months among people who inject drugs in Denver and Los Angeles (*n* = 472)

Factor	Adjusted Risk Ratio	95% confidence interval	*p*-value
Housing status			
Housed	Referent	-	-
Unhoused, not moved	2.75	1.00, 7.56	0.051
Unhoused, self-directed moves	4.54	1.68, 12.27	0.003
Unhoused, forcibly displaced	2.76	1.04, 7.29	0.041
Rushed injection in the last 3 months			
Not rushed	Referent	-	-
Rushed 1 to 9 times	1.45	0.86, 2.45	0.163
Rushed 10 or more times	2.09	1.32, 3.38	0.002
Any syringe reuse			
No	Referent	-	-
Yes	2.06	1.44, 2.94	< 0.001
Any goofball use in the last 3 months			
No	Referent	-	-
Yes	1.78	1.13, 2.81	0.014
Recruitment Location			
Denver	Referent	-	-
Los Angeles	0.79	0.53, 1.18	0.251

In a multivariable model that adjusted for confounding variables, including recruitment site (Table 5), all categories of being unhoused significantly increased risk of sharing either cookers/cottons (unhoused and not moved: RR = 2.26, 95% CI = 1.25, 4.06, *p* = 0.007; unhoused and self-directed moves: RR = 2.59, 95% CI = 1.41, 4.75, *p* = 0.002 and being unhoused and forcibly displaced: RR = 2.46, 95% CI = 1.40, 4.32, *p* = 0.002) as compared to housed participants.

**Table 5:** Binomial generalized linear model of cooker/cotton sharing in the last 3 months among people who inject drugs in Denver and Los Angeles (*N* = 472)

Factor	Adjusted Risk Ratio	95% confidence interval	*p*-value
Housing status			
Housed	Referent		
Unhoused, not moved	2.26	1.25, 4.06	0.006
Unhoused, self-directed moves	2.59	1.41, 4.75	0.002
Unhoused, forcibly displaced	2.46	1.40, 4.33	0.002
Rushed injection in the last 3 months			
Not rushed	Referent		
Rushed 1 to 9 times	1.43	1.07, 1.91	0.017
Rushed 10 or more times	1.56	1.18, 2.06	0.002
Any goofball use in the last 3 months			
No	Referent		
Yes	1.75	1.31, 2.35	< 0.001
Any opioid withdrawal symptoms in the last 3 months			
No	Referent		
Yes	1.98	1.34, 2.89	< 0.001
Recruitment Site			
Denver	Referent		
Los Angeles	1.06	0.87, 1.30	0.562

## Discussion

Our findings indicate that unhoused PWID in Los Angeles and Denver subject to forced displacement events are at heightened risk of negative health outcomes including nonfatal overdose, receptive syringe sharing, and sharing of injection-related materials. Additionally, while item confiscation and loss were common among all unhoused participants in this study, those with experience of forced displacement faced greater risk of confiscation of paperwork, medications, tents/shelters, and syringe disposal containers. These high rates of confiscation and disposal of material possessions among our participant population represent an added layer of vulnerability and potential health risk. However, our results also indicate that among these communities of PWID being unhoused, even when not subject to displacement, was associated with some of these same negative health risk outcomes. These results highlight the risk posed by the growing criminalization of homelessness—and specifically of homeless PWID—in the US and are especially relevant in light of the *City of Grants Pass v. Johnson* decision [15]. 

The prevalence of having material items confiscated and discarded among our participants is of concern given the extreme health vulnerability of homeless PWID. The increased risk experienced by displaced participants of losing items such as medications for opioid use disorder, biohazard containers for syringe storage, and tents/shelter materials indicates that government displacement is an inherently resource-depriving policy beyond the searches and seizures that homeless communities already experience under so-called “quality of life” laws [40]. While we were not able to include these variables in our main health outcome models due to variable structure and sample size constraints, we believe that the loss of these and other items creates further health vulnerability among this already socially and economically marginalized population. The increased risk of confiscation of medication, syringe storage containers, and basic shelter leaves homeless PWID more vulnerable to potential overdose, bloodborne infection risk, and skin and soft tissue infections.

The elevated risk of nonfatal overdose in association with forced displacement may be explained similarly. It is possible that these displacement events – and the complaint-oriented policing that often precedes them [20] - broke up local drug markets and moved participants into differing, less familiar drug markets and use environments. In this way, some self-directed moves and forced displacement events may produce similar outcomes to direct policing of drug markets [41–43]. These unfamiliar markets may have presented participants with drugs of unknown quality and potency (including unwanted fentanyl), as well as novel adulterants that could have heightened overdose potential. As these overdoses were non-fatal, we do not believe there was a confounding relationship with naloxone access, especially given that displacement was not associated with increased risk of naloxone confiscation.

Similarly, we do not believe there to be an association between syringe and injection supply access via syringe service programs (SSPs), syringe sharing, and government displacement. The majority (88%) of participants obtained syringes from an SSP in the past three months, and many of them were recruited at or near an SSP. Given the distribution and range of syringe service programs and other harm reduction services in Los Angeles and Denver, we assumed that displacement may have moved unhoused people who use drugs further away from these services. Multiple studies have shown that distance and travel time to these programs are predictors of health risk behaviors among PWID [44, 45]. However, in our sample we did not observe a significant association between past three month SSP utilization and movement or displacement.

The results of our analysis should be interpreted with several potential limitations in mind. As data presented here are cross-sectional, no causal relationships could be assessed. Stepwise regression also presents several limitations and biases that should be acknowledged. The results of stepwise regression may be severely impacted by collinearity, while regression coefficients may be biased high and confidence intervals biased to be narrow. We assessed collinearity of all variables under consideration for inclusion to address this potential issue but should acknowledge the potential bias in our results. Several forms of bias inherent to survey-based research may also have influenced collected data, including participant recall and social desirability bias, although measures similar to the ones used in this study have been found to be reliable and valid in other samples of people who inject drugs [26, 27]. Additionally, while the data collected was comprehensive with hundreds of survey items presented to participants, there are potentially unaccounted for confounders that may have influenced the magnitude of statistical associations. As the majority of our participants were recruited at or near SSPs, our data may have been biased towards those PWID who were able to access an SSP regardless of displacement experience. Future work examining displacement and resource access should aim to recruit a more geographically diverse sample.

## Conclusions

These results are situated within a large body of literature examining the multitude of risk environment characteristics that influence drug use behaviors and health outcomes. Our analysis indicates that displacement and communal breakup of unhoused people who use drugs, whether forced or self-directed, may influence health risk outcomes. As the number of unhoused individuals in the United States continues to grow, we believe that the implementation of policies of forcible and coerced displacement will contribute to increases in associated negative health outcomes. This potentially sets back much of the progress made by the success of harm reduction programming on overdose and new HIV and Hepatitis C infections, in addition to worsening the conditions that exacerbate skin and soft tissue infections [24]. Based on the outcomes of this emerging body of research, we encourage policymakers and governments to halt policies of forced displacement, and instead adopt alternative strategies including low-barrier, housing first models [46]. 

## Supplementary Information

Supplementary Material 1.

## Data Availability

The datasets generated and/or analyzed during the current study are not publicly available due to contents which could potentially identify research participants engaged in illegal activity, but are available from the corresponding author [JLG] and last author [RNB] on reasonable request.

## Abbreviations

HUD: Housing and Urban Development
PWID: People who inject drugs
RR: Risk Ratio
STD: Standard deviation
IQR: Interquartile range
CI: Confidence interval
SSP: Syringe services program
