# Supplementary material for: Housing and displacement as risk factors for negative health outcomes among people who inject drugs in Los Angeles, CA, and Denver, CO, USA
Source: BMC Public Health. 2026 Jan 31;26:753. doi: 10.1186/s12889-026-26200-2 (PMC12947454; doi:10.1186/s12889-026-26200-2)
Supplement: Supplementary file 1 — Supplementary Material 1. [file 12889_2026_26200_MOESM1_ESM.docx]

Supplemental File 1 – Baseline Questionnaire (English)

Q1. **Id number** __ __ __ __ __ __

Q2. **Site** (Choose one) 0 Denver

1 Los Angeles

Q3. **Interviewer initials (please record First, Middle, and Last Initial).** __ __ __

**A. BACKGROUND**

**First, thank you for agreeing to be interviewed. I just want to emphasize that everything you tell me will be kept confidential. Your honesty is critical to the success of this project and will be important in understanding how to improve community health. We will start by asking a few questions about your background.**

A1. **What is your date of birth?** __ __ / __ __ / __ __ __ __ mm / dd / yyyy

2097 Don't Know (Year)

2098 Refuse to Answer (Year)

A2. **How old are you?** __ __ __ Age

997 Don't Know

998 Refuse to Answer

999 Not Applicable

SUBAGE = (TODAY - A1)/365

A3. **Are you male, female, transgender, or gender non-conforming?** (Choose one)

0 Male

1 Female

2 Transgender

3 Gender non-conforming

4 Other

7 Don't Know

8 Refuse to Answer

If A3 is less than 2, then skip to A5.

If A3 is greater than 2, then skip to A5.

A4. **Are you a male to female or female to male transgender person?** (Choose one)

0 Male to female

1 Female to male

7 Don't Know

8 Refuse to Answer

9 Not Applicable

A5. **Do you consider yourself to be Hispanic or Latino?** 1 Yes

0 No

7 Don't Know

8 Refuse to Answer

9 Not Applicable

A6. **What is your race? (check all that apply; do not read list)** (Check all that apply)

__ White

__ Black

__ Asian

__ Pacific Islander

__ Native American or Alaskan Native

__ Mixed race

__ Other

__ Don't Know

__ Refuse to Answer

__ Not Applicable

A7. **In the past month, how much money did you make from all sources, including legal and illegal sources? READ LIST** (Choose one)

0 Less than $1,000

1 $1,000 to $1,400

2 $1,401 to $2,100

3 $2,101 or more

7 Don't Know

8 Refuse to Answer

A8. **In the last three months, did you receive income from:***(Read list aloud.)* (Check all that apply)

Job you had |__|

Unemployment |__|

VA benefits |__|

Welfare, food stamps, TANF, GA/GR |__|

SSDI-Supplemental Security Disability Income or State disability |__|

SSI or retirement benefits |__|

Family and/or spouse |__|

Recycling |__|

Panhandling |__|

Illegal or possibly illegal income |__|

Friend |__|

No income |__|

Don't Know |__|

Refuse to Answer |__|

A9. **Did you graduate from high school or get a GED?** 1 Yes

0 No

7 Don't Know

8 Refuse to Answer

9 Not Applicable

A10. **Have you ever served in the US Armed Forces?** 1 Yes

0 No

7 Don't Know

8 Refuse to Answer

9 Not Applicable

A11. **What is your current relationship status? (Read list, choose one)** (Choose one)

0 Single, no main partner

1 In relationship but not living as married

2 Married or living as married

7 Don't Know

8 Refuse to Answer

9 Not Applicable

A12. **In the last 3 months, have you been homeless or unstably housed?**

Yes 1

No 0 Skip to A14

Don't Know 7

Refuse to Answer 8

A13. **For how long have you been homeless or unstably housed? (Record as months - if less than a month enter 1)** (**That is, how long has it been since you were last stably housed.**)

__ __ __ __ Months

9997 Don't Know

9998 Refuse to Answer

9999 Not Applicable

A14. **What is the zip code of the area where you usually stay/sleep? (Refer to map if necessary)** (**If zipcode is unknown, please use the comment function (control-C) to record the street and nearest cross-street where the participant last slept.**)

__ __ __ __ __ Zip code

99997 Don't Know

99998 Refuse to Answer

99999 Not Applicable

A15. **In the last 3 months, let me know if you have stayed in any of the following types of places, even for one night?** (Check all that apply)

__ Tent

__ Outdoors (not in a tent)

__ Vehicle

__ Abandon building/garage/shed

__ Own house, apt, hotel room

__ Temporary hotel

__ Rented room in someone's place

__ With family, friend, girl/boyfriend

__ Shelter

__ Hospital

__ Jail or prison

__ Residential treatment

__ Group home

__ Don't Know

__ Refuse to Answer

__ Not Applicable

OUTDOOR = A15A + A15B + A15C + A15D

If OUTDOOR is equal to 0, then skip to instruction before A19.

A16. **In the last 3 months, how many nights did you stay in a tent, outdoors or in a car?**

__ __ Number

97 Don't Know

98 Refuse to Answer

99 Not Applicable

If A16 is less than 2, then skip to instruction before A19.

A17. **In the last 3 months, how many times did you move your tent, vehicle, or belongings to a different outdoor location (including across the street or down the block)?**

__ __ __ Moves

997 Don't Know

998 Refuse to Answer

999 Not Applicable

If A17 is equal to 0, then skip to instruction before A19.

A18. **In the last 3 months, how many of these moves were because of police, security guards, or other city/county officials?**

__ __

97 Don't Know

98 Refuse to Answer

99 Not Applicable

If A17 is less than A18 then READ: "Your government induced moves were more than you total moves. Please go back 2 questions and clarify." and skip to instruction before A18.

If A12 is equal to 0, then skip to A22.

A19. **In the last 3 months, have you had any of your belongings taken or discarded by city, state, or county employees such as police or sanitation workers?**

1 Yes

0 No

7 Don't Know

8 Refuse to Answer

9 Not Applicable

If A19 is equal to 0, then skip to A21.

A20. **In the last 3 months, what kind of items were taken or discarded by governmental officials? (READ LIST)** (Check all that apply)

__ Identification

__ Other important paper work

__ Naloxone/Narcan

__ Suboxone/subutex (buprenorphine)

__ Chronic condition medication (e.g. diabetes)

__ Acute condition medication (e.g., bacterial infection)

__ Clothing

__ Food

__ Illicit drugs

__ Photos

__ Pet(s)

__ Syringes

__ Cotton or cookers (paraphernalia)

__ Biohazard containers

__ Tent or shelter

__ Don't Know

__ Refuse to Answer

__ Not Applicable

A21. **In the last 3 months, has any outreach worker or case manager offered you any kind of housing (shelter, transitional housing, permanent housing)?** (**We want to know if you were offered an actually housing slot that you could move into, rather than being put on a waiting list.**)

1 Yes

0 No

7 Don't Know

8 Refuse to Answer

9 Not Applicable

A22. **In the last 3 months, have you been evicted or asked to leave housing that you considered permanent or semi-permanent? An example of semi-permanent housing is a person who is in 6 month transitional housing and gets evicted at the end of the 6 months without obtaining new permanent housing.**

1 Yes

0 No

7 Don't Know

8 Refuse to Answer

9 Not Applicable

**B. Substance use**

**The next questions are about your use of substances.**

B1. **Have you ever injected drugs?** 1 Yes

0 No

7 Don't Know

8 Refuse to Answer

9 Not Applicable

If B1 is equal to 0, then skip to end of questionnaire.

B2. **The first time you injected drugs, how old were you?** __ __ Age

97 Don't Know

98 Refuse to Answer

99 Not Applicable

B3. **What was the first drug that you used by injection? (Do not read list)** (Choose one)

00 Crack

01 Powder Cocaine

02 Heroin

03 Fentanyl or other synthetic opioid

04 Prescription opiates like oxycontin

05 Methamphetamine or amphetamine

06 Speedball (heroin & cocaine combined)

07 Goofball (heroin & meth combined)

08 Prescription stimulant like ritalin

09 Prescription tranquilizer/benzodiazepines

10 Cannabis product

11 Methadone

12 Buprenorphine

13 Sedative/barbiturates

14 Ketamine/dissociative

15 Other

97 Don't Know

98 Refuse to Answer

99 Not Applicable

If B3 is less than 15, then skip to B5.

B4. **What was the other drug that you injected first?**

__ __ __ __ __ __ __ __ __ __ __ __ __ __ __ __ __ __ __ __ __ __ __ __ __ __ __ __ __ __ __ __ __ __ __ __ __ __ __ __ __ __ __ __ __ __ __ __ __ __

B5. **Had you used the first drug injected by some other route of use (for example, swallowed, snorted or smoked) before injecting it the first time?**

1 Yes

0 No

7 Don't Know

8 Refuse to Answer

If B5 is equal to 0, then skip to B8.

B6. **How had you taken this drug prior to injecting it? (Check all that apply)** (Check all that apply)

__ Smoked it

__ Sniffed or snorted it

__ Inhaled or "chased" it

__ Swallowed it

__ Inserted it rectally/anally

__ Other

__ Don't Know

__ Refuse to Answer

__ Not Applicable

If B6F is equal to 0, then skip to B8.

B7. **What other way did you use this drug?**

__ __ __ __ __ __ __ __ __ __ __ __ __ __ __ __ __ __ __ __ __ __ __ __ __ __ __ __ __ __ __ __ __ __ __ __ __ __ __ __ __ __ __ __ __ __ __ __ __ __

B8. **Have you used speedball in the last 3 months (a mixture of heroin and cocaine or crack)?**

1 Yes

0 No

7 Don't Know

8 Refuse to Answer

9 Not Applicable

If B8 is equal to 0, then skip to B13.

B9. **In the last 3 months, how many times have you used a speedball without injecting?**

__ __ __ __ Times

9997 Don't Know

9998 Refuse to Answer

9999 Not Applicable

If B9 is equal to 0, then skip to B12.

B10. **For your non-injection speedball use, how did you use or ingest this combination? Check all that apply** (Check all that apply)

__ Smoked (burned)

__ Sniffed or snorted by nose

__ Inhaled by mouth (heated)

__ Swallowed

__ Sublingual or dissolved under tongue

__ Rectal/Anal

__ Other

__ Don't Know

__ Refuse to Answer

__ Not Applicable

If B10G is equal to 0, then skip to B12.

B11. **What other method did you use for ingesting a speedball?**

__ __ __ __ __ __ __ __ __ __ __ __ __ __ __ __ __ __ __ __ __ __ __ __ __ __ __ __ __ __ __ __ __ __ __ __ __ __ __ __ __ __ __ __ __ __ __ __ __ __ __ __ __ __ __ __ __ __ __ __

B12. **In the last 3 months, how many times have you injected a speedball?**

__ __ __ __ Times

9997 Don't Know

9998 Refuse to Answer

9999 Not Applicable

B13. **Have you used goofball in the last 3 months (a mixture of heroin and methamphetamine)?**

1 Yes

0 No

7 Don't Know

8 Refuse to Answer

9 Not Applicable

If B13 is equal to 0, then skip to B18.

B14. **In the last 3 months, how many times have you used a goofball without injecting?**

__ __ __ __ Times

9997 Don't Know

9998 Refuse to Answer

9999 Not Applicable

If B14 is equal to 0, then skip to B17.

B15. **For your non-injection goofball use, how did you use or ingest this combination? Check all that apply** (Check all that apply)

__ Smoked (burned)

__ Sniffed or snorted by nose

__ Inhaled by mouth (heated)

__ Swallowed

__ Sublingual or dissolved under tongue

__ Rectal/Anal

__ Other

__ Don't Know

__ Refuse to Answer

__ Not Applicable

If B15G is equal to 0, then skip to B17.

B16. **What other method did you use for ingesting a goofball?**

__ __ __ __ __ __ __ __ __ __ __ __ __ __ __ __ __ __ __ __ __ __ __ __ __ __ __ __ __ __ __ __ __ __ __ __ __ __ __ __ __ __ __ __ __ __ __ __ __ __ __ __ __ __ __ __ __ __ __ __

B17. **In the last 3 months, how many times have you injected a goofball?**

__ __ __ __ Times

9997 Don't Know

9998 Refuse to Answer

9999 Not Applicable

B18. **Have you used crack or crack cocaine in the last 3 months?** 1 Yes

0 No

7 Don't Know

8 Refuse to Answer

9 Not Applicable

If B18 is equal to 0, then skip to B23.

B19. **In the last 3 months, how many times have you used crack without injecting?**

__ __ __ __ Times

9997 Don't Know

9998 Refuse to Answer

9999 Not Applicable

If B19 is equal to 0, then skip to B22.

B20. **For your non-injection crack use, how did you use or ingest this drug? Check all that apply** (Check all that apply)

__ Smoked (burned)

__ Sniffed or snorted by nose

__ Inhaled by mouth (heated)

__ Swallowed

__ Sublingual or dissolved under tongue

__ Rectal/Anal

__ Other

__ Don't Know

__ Refuse to Answer

__ Not Applicable

If B20G is equal to 0, then skip to B22.

B21. **What other method did you use for ingesting a crack?**

__ __ __ __ __ __ __ __ __ __ __ __ __ __ __ __ __ __ __ __ __ __ __ __ __ __ __ __ __ __ __ __ __ __ __ __ __ __ __ __ __ __ __ __ __ __ __ __ __ __ __ __ __ __ __ __ __ __ __ __

B22. **In the last 3 months, how many times have you injected crack?** __ __ __ __ Times

9997 Don't Know

9998 Refuse to Answer

9999 Not Applicable

B23. **Have you used powder cocaine in the last 3 months?** 1 Yes

0 No

7 Don't Know

8 Refuse to Answer

9 Not Applicable

If B23 is equal to 0, then skip to B28.

B24. **In the last 3 months, how many times have you used powder cocaine without injecting?**

__ __ __ __ Times

9997 Don't Know

9998 Refuse to Answer

9999 Not Applicable

If B24 is equal to 0, then skip to B27.

B25. **For your non-injection powder cocaine use, how did you use or ingest this drug?** (Check all that apply)

__ Smoked (burned)

__ Sniffed or snorted by nose

__ Inhaled by mouth (heated)

__ Swallowed

__ Sublingual or dissolved under tongue

__ Rectal/Anal

__ Other

__ Don't Know

__ Refuse to Answer

__ Not Applicable

If B25G is equal to 0, then skip to B27.

B26. **What other method did you use for ingesting powder cocaine?**

__ __ __ __ __ __ __ __ __ __ __ __ __ __ __ __ __ __ __ __ __ __ __ __ __ __ __ __ __ __ __ __ __ __ __ __ __ __ __ __ __ __ __ __ __ __ __ __ __ __ __ __ __ __ __ __ __ __ __ __

B27. **In the last 3 months, how many times have you injected powder cocaine?**

__ __ __ __ Times

9997 Don't Know

9998 Refuse to Answer

9999 Not Applicable

B28. **Have you used methamphetamine by itself in the last 3 months?** 1 Yes

0 No

7 Don't Know

8 Refuse to Answer

9 Not Applicable

If B28 is equal to 0, then skip to B33.

B29. **In the last 3 months, how many times have you used methamphetamine by itself without injecting?**

__ __ __ __ Times

9997 Don't Know

9998 Refuse to Answer

9999 Not Applicable

If B29 is equal to 0, then skip to B32.

B30. **For your non-injection methamphetamine use, how did you use or ingest this drug?** (Check all that apply)

__ Smoked (burned)

__ Sniffed or snorted by nose

__ Inhaled by mouth (heated)

__ Swallowed

__ Sublingual or dissolved under tongue

__ Rectal/Anal

__ Other

__ Don't Know

__ Refuse to Answer

__ Not Applicable

If B30G is equal to 0, then skip to B32.

B31. **What other method did you use for ingesting methamphetamine?**

__ __ __ __ __ __ __ __ __ __ __ __ __ __ __ __ __ __ __ __ __ __ __ __ __ __ __ __ __ __ __ __ __ __ __ __ __ __ __ __ __ __ __ __ __ __ __ __ __ __ __ __ __ __ __ __ __ __ __ __

B32. **In the last 3 months, how many times have you injected methamphetamine by itself?**

__ __ __ __ Times

9997 Don't Know

9998 Refuse to Answer

9999 Not Applicable

B33. **Have you used heroin by itself in the last 3 months?** 1 Yes

0 No

7 Don't Know

8 Refuse to Answer

9 Not Applicable

If B33 is equal to 0, then skip to B38.

B34. **In the last 3 months, how many times have you used heroin without injecting?**

__ __ __ __ Times

9997 Don't Know

9998 Refuse to Answer

9999 Not Applicable

If B34 is equal to 0, then skip to B37.

B35. **For your non-injection heroin use, how did you use or ingest this drug?** (Check all that apply)

__ Smoked (burned)

__ Sniffed or snorted by nose

__ Inhaled by mouth (heated)

__ Swallowed

__ Sublingual or dissolved under tongue

__ Rectal/Anal

__ Other

__ Don't Know

__ Refuse to Answer

__ Not Applicable

If B35G is equal to 0, then skip to B37.

B36. **What other method did you use for ingesting heroin?**

__ __ __ __ __ __ __ __ __ __ __ __ __ __ __ __ __ __ __ __ __ __ __ __ __ __ __ __ __ __ __ __ __ __ __ __ __ __ __ __ __ __ __ __ __ __ __ __ __ __ __ __ __ __ __ __ __ __ __ __

B37. **In the last 3 months, how many times have you injected heroin by itself?**

__ __ __ __ Times

9997 Don't Know

9998 Refuse to Answer

9999 Not Applicable

B38. **Have you used fentanyl or another synthetic opioid (such as carfentanil) without a prescription in the last 3 months by itself?** (**Include synthetic opioids that were licitly (obtained from hospital or pharmacy) and illicitly manufactured drug.**)

1 Yes

0 No

7 Don't Know

8 Refuse to Answer

9 Not Applicable

If B38 is equal to 0, then skip to instruction before B43.

B39. **In the last 3 months, how many times have you used fentanyl without injecting by itself?**

__ __ __ __ Times

9997 Don't Know

9998 Refuse to Answer

9999 Not Applicable

If B39 is equal to 0, then skip to B42.

B40. **For your non-injection fentanyl use, how did you use or ingest it? Check all that apply** (Check all that apply)

__ Smoked it

__ Sniffed or snorted it

__ Inhaled or "chased" it

__ Swallowed it

__ Inserted it rectally

__ Used as patch

__ Other

__ Don't Know

__ Refuse to Answer

__ Not Applicable

If B40G is equal to 0, then skip to B42.

B41. **What other method did you use for ingesting fentanyl?**

__ __ __ __ __ __ __ __ __ __ __ __ __ __ __ __ __ __ __ __ __ __ __ __ __ __ __ __ __ __ __ __ __ __ __ __ __ __ __ __ __ __ __ __ __ __ __ __ __ __ __ __ __ __ __ __ __ __ __ __

B42. **In the last 3 months, how many times have you injected fentanyl by itself?**

__ __ __ __ Times

9997 Don't Know

9998 Refuse to Answer

9999 Not Applicable

**The next set of questions is about drugs that are typically prescribed by physicians. We are interested in your use of these medications without a doctor's prescription or your use of these medications not as directed by your physician.**

B43. **Have you used an opioid (such as tramadol, vicodin, oxycontin, opana, dilaudid, percocet, oxycodone, or morphine) without a prescription in the last 3 months?** (**Do not include synthetic opioids like fentanyl or carfentanil**)

1 Yes

0 No

7 Don't Know

8 Refuse to Answer

9 Not Applicable

If B43 is equal to 0, then skip to B48.

B44. **In the last 3 months, how many times have you used an opioid without injecting?**

__ __ __ __ Times

9997 Don't Know

9998 Refuse to Answer

9999 Not Applicable

If B44 is equal to 0, then skip to B47.

B45. **For your non-injection prescription opioid use, how did you use or ingest this drug?** (Check all that apply)

__ Smoked it

__ Sniffed or snorted it

__ Inhaled or "chased" it

__ Swallowed it

__ Inserted it rectally

__ Other

__ Don't Know

__ Refuse to Answer

__ Not Applicable

If B45F is equal to 0, then skip to B47.

B46. **What other method did you use for ingesting opioid prescription medications?**

__ __ __ __ __ __ __ __ __ __ __ __ __ __ __ __ __ __ __ __ __ __ __ __ __ __ __ __ __ __ __ __ __ __ __ __ __ __ __ __ __ __ __ __ __ __ __ __ __ __ __ __ __ __ __ __ __ __ __ __

B47. **In the last 3 months, how many times have you injected an opioid?**

__ __ __ __ Times

9997 Don't Know

9998 Refuse to Answer

9999 Not Applicable

B48. **Have you used a tranquilizer/benzodiazepine (such as klonopin, Xanax, valium, or ativan) without a prescription in the last 3 months?** (Or research chemicals such as clonzolam, bromazolam, and flualprazolam)

1 Yes

0 No

7 Don't Know

8 Refuse to Answer

9 Not Applicable

If B48 is equal to 0, then skip to B53.

B49. **In the last 3 months, how many times have you used a tranquilizer/benzodiazepine without injecting?**

__ __ __ __ Times

9997 Don't Know

9998 Refuse to Answer

9999 Not Applicable

If B49 is equal to 0, then skip to B52.

B50. **For your non-injection tranquilizer/benzodiazepine use, how did you use or ingest it? Check all that apply** (Check all that apply)

__ Smoked (burned)

__ Sniffed or snorted by nose

__ Inhaled by mouth (heated)

__ Swallowed

__ Sublingual or dissolved under tongue

__ Rectal/Anal

__ Other

__ Don't Know

__ Refuse to Answer

__ Not Applicable

If B50G is equal to 0, then skip to B52.

B51. **What other method did you use for ingesting a TRANQUILIZER/benzodiazepine?**

__ __ __ __ __ __ __ __ __ __ __ __ __ __ __ __ __ __ __ __ __ __ __ __ __ __ __ __ __ __ __ __ __ __ __ __ __ __ __ __ __ __ __ __ __ __ __ __ __ __ __ __ __ __ __ __ __ __ __ __

B52. **In the last 3 months, how many times have you injected tranquilizers/benzodiazepines?**

__ __ __ __ Times

9997 Don't Know

9998 Refuse to Answer

9999 Not Applicable

B53. **Have you used a sedative (such as restoril, phenobarbital, placidyl, ambien, Lunesta, gabapentin) without a prescription in the last 3 months?**

1 Yes

0 No

7 Don't Know

8 Refuse to Answer

9 Not Applicable

If B53 is equal to 0, then skip to B58.

B54. **In the last 3 months, how many times have you used a sedative without injecting?**

__ __ __ __ Times

9997 Don't Know

9998 Refuse to Answer

9999 Not Applicable

If B54 is equal to 0, then skip to B57.

B55. **For your non-injection sedative use, how did you use or ingest it? Check all that apply** (Check all that apply)

__ Smoked (burned)

__ Sniffed or snorted by nose

__ Inhaled by mouth (heated)

__ Swallowed

__ Sublingual or dissolved under tongue

__ Rectal/Anal

__ Other

__ Don't Know

__ Refuse to Answer

__ Not Applicable

If B55G is equal to 0, then skip to B57.

B56. **What other method did you use for ingesting a sedative?**

__ __ __ __ __ __ __ __ __ __ __ __ __ __ __ __ __ __ __ __ __ __ __ __ __ __ __ __ __ __ __ __ __ __ __ __ __ __ __ __ __ __ __ __ __ __ __ __ __ __ __ __ __ __ __ __ __ __ __ __

B57. **In the last 3 months, how many times have you injected a sedative?**

__ __ __ __ Times

9997 Don't Know

9998 Refuse to Answer

9999 Not Applicable

B58. **Have you used a stimulant (such as ritalin, aderall, methedrine, benzedrine) without a prescription in the last 3 months?**

1 Yes

0 No

7 Don't Know

8 Refuse to Answer

9 Not Applicable

If B58 is equal to 0, then skip to B63.

B59. **In the last 3 months, how many times have you used a stimulant without injecting?**

__ __ __ __ Times

9997 Don't Know

9998 Refuse to Answer

9999 Not Applicable

If B59 is equal to 0, then skip to B62.

B60. **For your non-injection stimulant use, how did you use or ingest it? Check all that apply** (Check all that apply)

__ Smoked (burned)

__ Sniffed or snorted by nose

__ Inhaled by mouth (heated)

__ Swallowed

__ Sublingual or dissolved under tongue

__ Rectal/Anal

__ Other

__ Don't Know

__ Refuse to Answer

__ Not Applicable

If B60G is equal to 0, then skip to B62.

B61. **What other method did you use for ingesting a stimulant?**

__ __ __ __ __ __ __ __ __ __ __ __ __ __ __ __ __ __ __ __ __ __ __ __ __ __ __ __ __ __ __ __ __ __ __ __ __ __ __ __ __ __ __ __ __ __ __ __ __ __ __ __ __ __ __ __ __ __ __ __

B62. **In the last 3 months, how many times have you injected a stimulant?**

__ __ __ __ Times

9997 Don't Know

9998 Refuse to Answer

9999 Not Applicable

B63. **Have you used a methadone without a prescription in the last 3 months?** 1 Yes

0 No

7 Don't Know

8 Refuse to Answer

9 Not Applicable

If B63 is equal to 0, then skip to B68.

B64. **In the last 3 months, how many times have you used methadone without injecting?**

__ __ __ __ Times

9997 Don't Know

9998 Refuse to Answer

9999 Not Applicable

If B64 is equal to 0, then skip to B67.

B65. **For your non-injection methadone use, how did you use or ingest it? Check all that apply** (Check all that apply)

__ Smoked (burned)

__ Sniffed or snorted by nose

__ Inhaled by mouth (heated)

__ Swallowed

__ Sublingual or dissolved under tongue

__ Rectal/Anal

__ Other

__ Don't Know

__ Refuse to Answer

__ Not Applicable

If B65G is equal to 0, then skip to B67.

B66. **What other method did you use for ingesting methadone?**

__ __ __ __ __ __ __ __ __ __ __ __ __ __ __ __ __ __ __ __ __ __ __ __ __ __ __ __ __ __ __ __ __ __ __ __ __ __ __ __ __ __ __ __ __ __ __ __ __ __ __ __ __ __ __ __ __ __ __ __

B67. **In the last 3 months, how many times have you injected methadone?**

__ __ __ __ Times

9997 Don't Know

9998 Refuse to Answer

9999 Not Applicable

B68. **Have you used buprenorphine, suboxone, or subutex without a prescription in the last 3 months?**

1 Yes

0 No

7 Don't Know

8 Refuse to Answer

9 Not Applicable

If B68 is equal to 0, then skip to instruction before B73.

B69. **In the last 3 months, how many times have you used buprenorphine without injecting?**

__ __ __ __ Times

9997 Don't Know

9998 Refuse to Answer

9999 Not Applicable

If B69 is equal to 0, then skip to B72.

B70. **For your non-injection buprenorphine use, how did you use or ingest it? Check all that apply** (Check all that apply)

__ Smoked (burned)

__ Sniffed or snorted by nose

__ Inhaled by mouth (heated)

__ Swallowed

__ Sublingual or dissolved under tongue

__ Rectal/Anal

__ Other

__ Don't Know

__ Refuse to Answer

__ Not Applicable

If B70G is equal to 0, then skip to B72.

B71. **What other method did you use for ingesting buprenorphine?**

__ __ __ __ __ __ __ __ __ __ __ __ __ __ __ __ __ __ __ __ __ __ __ __ __ __ __ __ __ __ __ __ __ __ __ __ __ __ __ __ __ __ __ __ __ __ __ __ __ __ __ __ __ __ __ __ __ __ __ __

B72. **In the last 3 months, how many times have you injected buprenorphine?**

__ __ __ __ Times

9997 Don't Know

9998 Refuse to Answer

9999 Not Applicable

SUMINJ = B12 + B17 + B22 + B27 + B32 + B37 + B47 + B42 + B52 + B57 + B62 + B67 + B72

HERSUM = B9 + B12 + B14 + B17 + B34 + B37 + B44 + B47 + B39 + B42

COKSUM = B9 + B12 + B19 + B22 + B24 + B27

METSUM = B14 + B17 + B29 + B32

OSTSUM = B64 + B67 + B69 + B72

NUMOPIOID = B8 + B13 + B33 + B43 + B38

If NUMOPIOID is equal to 0, then skip to end of questionnaire.

**The next questions are about your use of marijuana and/or cannabis and cannabis products. We are interested in your use of marijuana/cannabis regardless of whether your use is for medical or non-medical purposes.**

B73. **Have you ever used marijuana or cannabis?** 1 Yes

0 No

7 Don't Know

8 Refuse to Answer

9 Not Applicable

If B73 is equal to 0, then skip to instruction before B116.

B74. **How old were you when you first used marijuana or cannabis?** __ __ Age

97 Don't Know

98 Refuse to Answer

99 Not Applicable

B75. **Have you used marijuana or cannabis in the last 3 months?** 1 Yes

0 No

7 Don't Know

8 Refuse to Answer

9 Not Applicable

If B75 is equal to 0, then skip to instruction before B116.

B76. **In the last 3 months, how many times have you used marijuana or cannabis?**

__ __ __ __ Times

9997 Don't Know

9998 Refuse to Answer

9999 Not Applicable

B77. **For your marijuana and cannabis use in the past 3 months, how did you use or ingest it? (DO NOT READ LIST)** (Check all that apply)

__ Smoked (burned/lit)

__ Vaped (heated)

__ Ate (Edibles)

__ Dab rig for concentrates/budder/badder

__ Topicals (cream, salve, oils)

__ Other oral use (tinctures, oils, capsules)

__ Injection

__ Other

__ Don't Know

__ Refuse to Answer

__ Not Applicable

If B77H is equal to 0, then skip to B79.

B78. **What other method did you use for ingesting marijuana or cannabis?**

__ __ __ __ __ __ __ __ __ __ __ __ __ __ __ __ __ __ __ __ __ __ __ __ __ __ __ __ __ __ __ __ __ __ __ __ __ __ __ __ __ __ __ __ __ __ __ __ __ __ __ __ __ __ __ __ __ __ __ __

B79. **How have you mostly used cannabis?**  (Choose one) 00 Smoked

01 Vaped

02 Ate

03 Dab rig (concentrate)

04 Topical cream/salve

05 Other oral

06 Injection

07 Other

97 Don't Know

98 Refuse to Answer

99 Not Applicable

SUMNON = B9 + B14 + B19 + B24 + B29 + B34 + B44 + B39 + B49 + B54 + B59 + B64 + B69 + B76

B80. **Do you have a medical marijuana/cannabis card or a referral letter for medical use of marijuana or cannabis?**

1 Yes

0 No

7 Don't Know

8 Refuse to Answer

9 Not Applicable

B81. **From which of the following sources have you obtained cannabis or marijuana from in the last 3 months? (Check all that apply)** (Check all that apply)

__ Friend or acquaintance

__ Family

__ Dealer (not dispensary)

__ Medical dispensary

__ Recreational dispensary

__ Substance use treatment program

__ Grew my own

__ Received from cannabis distribution program

__ Other

__ Don't Know

__ Refuse to Answer

__ Not Applicable

If B81I is equal to 0, then skip to B83.

B82. **What was this other source?** __ __ __ __ __ __ __ __ __ __ __ __ __ __ __ __ __ __ __ __

B83. **From which source did you obtain most of the cannabis you used in the last 3 months?**  (Choose one)

00 Friend or acquaintance

01 Family

02 Street dealer

03 Medical dispensary

04 Recreational dispensary

05 Substance use treatment clinic

06 Grew my own

07 Free cannabis distribution

08 Other

97 Don't Know

98 Refuse to Answer

99 Not Applicable

B84. **Which types of cannabis have you used in the last 3 months? (Read list)** (Check all that apply)

__ Indica flower

__ Sativa flower

__ Hybrid

__ High CBD

__ High THC

__ Concentrate

__ Other

__ Don't Know

__ Refuse to Answer

__ Not Applicable

If B84G is equal to 0, then skip to B86.

If B84G is equal to 97, then skip to B87.

B85. **What is this other type of cannabis that you used?**

__ __ __ __ __ __ __ __ __ __ __ __ __ __ __ __ __ __ __ __ __ __ __ __ __ __ __ __ __ __ __ __ __ __ __ __ __ __ __ __

B86. **Which type of cannabis did you use the most in the last 3 months** (Choose one)

0 Indica

1 Sativa

2 Hybrid

3 High CBD

4 High THC

5 Concentrate

6 Other

7 Don't Know

8 Refuse to Answer

9 Not Applicable

B87. **Have you purchased cannabis/marijuana products in the last 3 months?** 1 Yes

0 No

7 Don't Know

8 Refuse to Answer

9 Not Applicable

If B87 is equal to 0, then skip to instruction before B97.

If B81C is equal to 0, then skip to instruction before B91.

B88. **The last time you purchased cannabis from a source other than a dispensary, how much did the cannabis cost per gram? (dollars per gram)** (**For interviewer only: A dime bag of cannabis is usually a half gram and cost $10. So the gram cost of a dime bag in this example is $20. There are 28 grams in an ounce. Therefore, a 1/4 ounce is 7 grams. A typical joint is a third of a gram. So if a joint cost a dollar than the price per gram was $3.** )

$__ __ __ __ Dollars

9997 Don't Know

9998 Refuse to Answer

9999 Not Applicable

B89. **Was the amount you paid to this non-dispensary source more, less or about the same as you would usually spend for the same amount of cannabis/marijuana from this source?**  (Choose one)

0 Less

1 About the same

2 More

3 Don't purchase MJ from this source

7 Don't Know

8 Refuse to Answer

9 Not Applicable

B90. **How would you rate the quality of the cannabis/marijuana you last purchased from the dealer?** (Choose one)

0 Low grade

1 Medium grade

2 High grade

7 Don't Know

8 Refuse to Answer

9 Not Applicable

If B81D is equal to 0, then skip to instruction before B94.

B91. **The last time you purchased cannabis from a medical dispensary, how much did the cannabis cost per gram? (Dollars per gram)** (**For interviewer only: A dime bag of cannabis is usually a half gram and cost $10. So the gram cost of a dime bag in this example is $20. There are 28 grams in an ounce. Therefore, a 1/4 ounce is 7 grams. A typical joint is a third of a gram. So if a joint cost a dollar than the price per gram was $3.**
)

$__ __ __ __ Dollars

9997 Don't Know

9998 Refuse to Answer

9999 Not Applicable

B92. **Was the amount you paid to the medical dispensary more, less or about the same as you would usually spend for the same amount of cannabis/marijuana from this source?**  (Choose one)

0 Less

1 About the same

2 More

3 Don't purchase MJ from this source

7 Don't Know

8 Refuse to Answer

9 Not Applicable

B93. **How would you rate the quality of the cannabis/marijuana you last purchased from the medical dispensary?** (Choose one)

0 Low grade

1 Medium grade

2 High grade

7 Don't Know

8 Refuse to Answer

9 Not Applicable

If B81E is equal to 0, then skip to instruction before B97.

B94. **The last time you purchased cannabis from a recreational dispensary, how much did the cannabis cost per gram? (Dollars per gram)** (**For interviewer only: A dime bag of cannabis is usually a half gram and cost $10. So the gram cost of a dime bag in this example is $20. There are 28 grams in an ounce. Therefore, a 1/4 ounce is 7 grams. A typical joint is a third of a gram. So if a joint cost a dollar than the price per gram was $3.**
)

$__ __ __ __ Dollars

9997 Don't Know

9998 Refuse to Answer

9999 Not Applicable

B95. **Was the amount you paid to the recreational dispensary more, less or about the same as you would usually spend for the same amount of cannabis/marijuana from this source?**  (Choose one)

0 Less

1 About the same

2 More

3 Don't purchase MJ from this source

7 Don't Know

8 Refuse to Answer

9 Not Applicable

B96. **How would you rate the quality of the cannabis/marijuana you last purchased from the recreational dispensary?** (Choose one)

0 Low grade

1 Medium grade

2 High grade

7 Don't Know

8 Refuse to Answer

9 Not Applicable

**Many people drink alcohol when using cannabis. For the next 4 questions, we want to know how often you combine cannabis use with use of other substances.**

B97. **When you used marijuana or cannabis in the last 3 months, how often did you use it with alcohol?** (Choose one)

0 None

1 Some

2 About half

3 Most

4 All

7 Don't Know

8 Refuse to Answer

9 Not Applicable

B98. **When you used marijuana or cannabis in the last 3 months, how often did you use it with tobacco or nicotine?** (Choose one)

0 None

1 Some

2 About half

3 Most

4 All

7 Don't Know

8 Refuse to Answer

9 Not Applicable

B99. **When you used marijuana or cannabis in the last 3 months, how often did you use it with substances like heroin, methamphetamine, crack cocaine, cocaine?** (Choose one)

0 None

1 Some

2 About half

3 Most

4 All

7 Don't Know

8 Refuse to Answer

9 Not Applicable

If B99 is equal to 0, then skip to instruction before B103.

B100. **What other drug have you used the most with cannabis in the last 3 months? (Do not read list)** (Choose one)

00 Crack

01 Powder Cocaine

02 Heroin

03 Fentanyl or other synthetic opioid

04 Prescription opiates like oxycontin

05 Methamphetamine or amphetamine

06 Speedball (heroin & cocaine combined)

07 Goofball (heroin & meth combined)

08 Prescription stimulant like ritalin

09 Prescription tranquilizer/benzodiazepines

10 Methadone

11 Buprenorphine

12 Sedative/barbiturates

13 Other

97 Don't Know

98 Refuse to Answer

99 Not Applicable

B101. **In the last 3 months, when you have mixed cannabis with drugs like heroin, prescription opiates, methamphetamine, and cocaine, did you do this for any of the following reasons?** (Check all that apply)

__ To get a better high

__ To reduce the use of either or both substances

__ Because you had both

__ To modify or manage the high

__ To manage the come down

__ Other

__ Don't Know

__ Refuse to Answer

__ Not Applicable

If B101F is equal to 0, then skip to instruction before B103.

B102. **What were your other reasons for using cannabis with other substances?**

__ __ __ __ __ __ __ __ __ __ __ __ __ __ __ __ __ __ __ __ __ __ __ __ __ __ __ __ __ __ __ __ __ __ __ __ __ __ __ __

**The next set of questions are about your reasons for using cannabis in general.**

B103. **When you used marijuana or cannabis in the last 3 months, how often did you use it to relieve physical pain?** (Choose one)

0 None

1 Some

2 About half

3 Most

4 All

7 Don't Know

8 Refuse to Answer

9 Not Applicable

B104. **When you used marijuana or cannabis in the last 3 months, how often did you use it to relieve opioid or heroin withdrawal symptoms and/or pain?** (Choose one)

0 None

1 Some

2 About half

3 Most

4 All

7 Don't Know

8 Refuse to Answer

9 Not Applicable

B105. **When you used marijuana or cannabis in the last 3 months, how often did you use it to relieve emotional problems such as depression, anxiety, or feelings of sadness?** (Choose one)

0 None

1 Some

2 About half

3 Most

4 All

7 Don't Know

8 Refuse to Answer

9 Not Applicable

B106. **When you used marijuana or cannabis in the last 3 months, how often did you use it to reduce your heroin, fentanyl, or prescription opiate use?** (Choose one)

0 None

1 Some

2 About half

3 Most

4 All

7 Don't Know

8 Refuse to Answer

9 Not Applicable

B107. **When you used marijuana or cannabis in the last 3 months, how often did you use it to reduce your alcohol use?** (Choose one)

0 None

1 Some

2 About half

3 Most

4 All

7 Don't Know

8 Refuse to Answer

9 Not Applicable

B108. **When you used marijuana or cannabis in the last 3 months, how often did you use it to reduce your stimulant or methamphetamine use?** (Choose one)

0 None

1 Some

2 About half

3 Most

4 All

7 Don't Know

8 Refuse to Answer

9 Not Applicable

B109. **When you used marijuana or cannabis in the last 3 months, how often did you use it to help with sleep?** (Choose one)

0 None

1 Some

2 About half

3 Most

4 All

7 Don't Know

8 Refuse to Answer

9 Not Applicable

B110. **When you used marijuana or cannabis in the last 3 months, how often did you use it to treat nausea or loss of appetite?** (Choose one)

0 None

1 Some

2 About half

3 Most

4 All

7 Don't Know

8 Refuse to Answer

9 Not Applicable

B111. **When you used marijuana or cannabis in the last 3 months, how often did you use it to get high?** (Choose one)

0 None

1 Some

2 About half

3 Most

4 All

7 Don't Know

8 Refuse to Answer

9 Not Applicable

B112. **Have you EVER used cannabis/marijuana to reduce your use of other substances such as alcohol, tobacco, heroin, prescription opiates, etc?**

1 Yes

0 No

7 Don't Know

8 Refuse to Answer

9 Not Applicable

If B112 is equal to 0, then skip to instruction before B116.

B113. **Which drugs were you trying to reduce when you used cannabis?** (Check all that apply)

__ Alcohol

__ Tobacco or nicotine

__ Crack cocaine

__ Powder cocaine

__ Heroin

__ Fentanyl

__ Prescription opiates

__ Methamphetamine

__ Speedball

__ Goofball

__ Prescription stimulants

__ Prescription tranquilizers

__ Prescription sedatives

__ Methadone

__ Buprenorphine

__ Don't Know

__ Refuse to Answer

__ Not Applicable

B114. **Did it work for any drug?** 1 Yes

0 No

7 Don't Know

8 Refuse to Answer

9 Not Applicable

B115. **For which drug did using cannabis/marijuana result in a reduction in use?** (Check all that apply)

__ Alcohol

__ Tobacco or nicotine

__ Crack cocaine

__ Powder cocaine

__ Heroin

__ Fentanyl

__ Prescription opiates

__ Methamphetamine

__ Speedball

__ Goofball

__ Prescription stimulants

__ Prescription tranquilizers

__ Prescription sedatives

__ Methadone

__ Buprenorphine

__ Don't Know

__ Refuse to Answer

__ Not Applicable

**The next set of questions are about your use of alcohol and tobacco.**

B116. **In the last 3 months, on how many days did you drink a beverage containing alcohol?**

__ __ Days

97 Don't Know

98 Refuse to Answer

99 Not Applicable

If B116 is equal to 0, then skip to B119.

**For the next two items, please note that a standard alcohol drink is equal to 12 ounces of regular beer, 8 to 9 ounces of malt liquor beer, 5 ounces of wine, 3 to 4 ounces of fortified wine, brandy, or port, and 1.5 ounces of hard liquor (or a shot).**

B117. **During the last 3 months, on a typical day when you drank alcohol, how many standard drinks did you have?**

__ __ __ Drinks

997 Don't Know

998 Refuse to Answer

999 Not Applicable

B118. **In the last 3 months, what is the largest number of standard drinks you had on any single day?**

__ __ drinks

97 Don't Know

98 Refuse to Answer

99 Not Applicable

B119. **Do you currently smoke cigarettes? (Limit to combustible tobacco including cigars and pipes, but not e-cigarettes).**

1 Yes

0 No

7 Don't Know

8 Refuse to Answer

9 Not Applicable

If B119 is equal to 0, then skip to instruction before B122.

B120. **How many cigarettes do you smoke a day on average? (FYI, a pack has 20 cigarettes).**

__ __ __ # of cigarettes

997 Don't Know

998 Refuse to Answer

999 Not Applicable

B121. **How many years have you smoked?** __ __ Years

97 Don't Know

98 Refuse to Answer

99 Not Applicable

If B119 is equal to 1, then skip to instruction before B125.

B122. **Have you ever smoked cigarettes?** 1 Yes

0 No

7 Don't Know

8 Refuse to Answer

9 Not Applicable

If B122 is equal to 0, then skip to instruction before B125.

B123. **How many years did you smoke? (If less than a year, enter 1).** __ __ Years

97 Don't Know

98 Refuse to Answer

99 Not Applicable

B124. **How many packs a day did you smoke? (If less than 1 pack, enter 1) (a pack has 20 cigarettes).**

__ __ __

997 Don't Know

998 Refuse to Answer

999 Not Applicable

**The next questions are about whether fentanyl is available in illicit drug markets where you live and/or hang out. I am interested in fentanyl that you have obtained on the streets or illicitly and NOT that was prescribed to you.**

B125. **Is fentanyl (in any form) available in the area where you live and/or spend most of your time?** (**Any form includes only or mixed intentionally or unintentionally in other substances.**)

1 Yes

0 No

7 Don't Know

8 Refuse to Answer

9 Not Applicable

If B125 is equal to 0, then skip to B127.

B126. **In what form is fentanyl available? (READ LIST)** (Check all that apply)

__ Mixed with heroin/sold as mixed

__ Mixed with heroin/sold as heroin

__ Sold as fentanyl

__ Sold as heroin, but is only fentanyl

__ Sold as counterfeit pills w/out fentanyl

__ Sold as counterfeit pills with fentanyl

__ Don't Know

__ Refuse to Answer

__ Not Applicable

B127. **In the last 3 month, have you used any drug that you suspected or thought was mixed with fentanyl or some other synthetic opioid?**

1 Yes

0 No

7 Don't Know

8 Refuse to Answer

9 Not Applicable

If B127 is equal to 0, then skip to B143.

B128. **Please select the drugs that you used in the last 3 months that were mixed with fentanyl or other synthetic opioids?** (Check all that apply)

__ Crack

__ Powder cocaine

__ Heroin

__ Prescription Opiates

__ Methamphetamine

__ Speedball

__ Goofball

__ Stimulants

__ Tranquilizers

__ Cannabis

__ Methadone

__ Buprenorphine

__ Sedative

__ Ketamine/dissociative

__ Don't Know

__ Refuse to Answer

__ Not Applicable Skip to B143

If B128A is equal to 0, then skip to instruction before B130.

B129. **In the last 3 months, how frequently was the crack that you used mixed with fentanyl?** (Choose one)

0 Never - 0%

1 Occasionally - 1% to 25%

2 Sometimes - 26% to 74%

3 Usually - 75% to 99%

4 Always - 100%

7 Don't Know

8 Refuse to Answer

9 Not Applicable

If B128B is equal to 0, then skip to instruction before B131.

B130. **In the last 3 months, how frequently was the powder cocaine that you used mixed with fentanyl?** (Choose one)

0 Never - 0%

1 Occasionally - 1% to 25%

2 Sometimes - 26% to 74%

3 Usually - 75% to 99%

4 Always - 100%

7 Don't Know

8 Refuse to Answer

9 Not Applicable

If B128C is equal to 0, then skip to instruction before B132.

B131. **In the last 3 months, how frequently was the heroin you used mixed with fentanyl?** (Choose one)

0 Never - 0%

1 Occasionally - 1% to 25%

2 Sometimes - 26% to 74%

3 Usually - 75% to 99%

4 Always - 100%

7 Don't Know

8 Refuse to Answer

9 Not Applicable

If B128D is equal to 0, then skip to instruction before B133.

B132. **In the last 3 months, how frequently was the prescription opioids that you used mixed with fentanyl?** (Choose one)

0 Never - 0%

1 Occasionally - 1% to 25%

2 Sometimes - 26% to 74%

3 Usually - 75% to 99%

4 Always - 100%

7 Don't Know

8 Refuse to Answer

9 Not Applicable

If B128E is equal to 0, then skip to instruction before B134.

B133. **In the last 3 months, how frequently was the methamphetamine that you used mixed with fentanyl?** (Choose one)

0 Never - 0%

1 Occasionally - 1% to 25%

2 Sometimes - 26% to 74%

3 Usually - 75% to 99%

4 Always - 100%

7 Don't Know

8 Refuse to Answer

9 Not Applicable

If B128F is equal to 0, then skip to instruction before B135.

B134. **In the last 3 months, how frequently were the speedballs that you used mixed with fentanyl?** (Choose one)

0 Never - 0%

1 Occasionally - 1% to 25%

2 Sometimes - 26% to 74%

3 Usually - 75% to 99%

4 Always - 100%

7 Don't Know

8 Refuse to Answer

9 Not Applicable

If B128G is equal to 0, then skip to instruction before B136.

B135. **In the last 3 months, how frequently were the goofballs that you used mixed with fentanyl?** (Choose one)

0 Never - 0%

1 Occasionally - 1% to 25%

2 Sometimes - 26% to 74%

3 Usually - 75% to 99%

4 Always - 100%

7 Don't Know

8 Refuse to Answer

9 Not Applicable

If B128H is equal to 0, then skip to instruction before B137.

B136. **In the last 3 months, how frequently were the prescription stimulants that you used mixed with fentanyl?** (Choose one)

0 Never - 0%

1 Occasionally - 1% to 25%

2 Sometimes - 26% to 74%

3 Usually - 75% to 99%

4 Always - 100%

7 Don't Know

8 Refuse to Answer

9 Not Applicable

If B128I is equal to 0, then skip to instruction before B138.

B137. **In the last 3 months, how frequently were the prescription tranquilizers that you used mixed with fentanyl?** (Choose one)

0 Never - 0%

1 Occasionally - 1% to 25%

2 Sometimes - 26% to 74%

3 Usually - 75% to 99%

4 Always - 100%

7 Don't Know

8 Refuse to Answer

9 Not Applicable

If B128J is equal to 0, then skip to instruction before B139.

B138. **In the last 3 months, how frequently was the cannabis that you used mixed with fentanyl?** (Choose one)

0 Never - 0%

1 Occasionally - 1% to 25%

2 Sometimes - 26% to 74%

3 Usually - 75% to 99%

4 Always - 100%

7 Don't Know

8 Refuse to Answer

9 Not Applicable

If B128K is equal to 0, then skip to instruction before B140.

B139. **In the last 3 months, how frequently was the methadone that you used mixed with fentanyl?** (Choose one)

0 Never - 0%

1 Occasionally - 1% to 25%

2 Sometimes - 26% to 74%

3 Usually - 75% to 99%

4 Always - 100%

7 Don't Know

8 Refuse to Answer

9 Not Applicable

If B128L is equal to 0, then skip to instruction before B141.

B140. **In the last 3 months, how frequently was the buprenorphine/suboxone that you used mixed with fentanyl?** (Choose one)

0 Never - 0%

1 Occasionally - 1% to 25%

2 Sometimes - 26% to 74%

3 Usually - 75% to 99%

4 Always - 100%

7 Don't Know

8 Refuse to Answer

9 Not Applicable

If B128M is equal to 0, then skip to instruction before B142.

B141. **In the last 3 months, how frequently were the prescription sedatives that you used mixed with fentanyl?** (Choose one)

0 Never - 0%

1 Occasionally - 1% to 25%

2 Sometimes - 26% to 74%

3 Usually - 75% to 99%

4 Always - 100%

7 Don't Know

8 Refuse to Answer

9 Not Applicable

If B128N is equal to 0, then skip to B143.

B142. **In the last 3 months, how frequently was the ketamine/dissociative that you used mixed with fentanyl?** (Choose one)

0 Never - 0%

1 Occasionally - 1% to 25%

2 Sometimes - 26% to 74%

3 Usually - 75% to 99%

4 Always - 100%

7 Don't Know

8 Refuse to Answer

9 Not Applicable

B143. **Have you ever used a fentanyl test strip on drugs you were about to use? (either injected or non-injected).**

1 Yes

0 No

7 Don't Know

8 Refuse to Answer

9 Not Applicable

If B143 is equal to 0, then skip to instruction before C1.

B144. **How many times have use ever used a fentanyl test strip to check drugs you were about to use?**

__ __ __ __ __ Times

99997 Don't Know

99998 Refuse to Answer

99999 Not Applicable

B145. **Have you used a fentanyl test strip in the last 3 months?** 1 Yes

0 No

7 Don't Know

8 Refuse to Answer

9 Not Applicable

B146. **The last time you used a fentanyl test strip, was there fentanyl in the sample?**

1 Yes

0 No

7 Don't Know

8 Refuse to Answer

9 Not Applicable

If B146 is equal to 0, then skip to B148.

B147. **What did you do following the positive fentanyl test result?** (Check all that apply)

__ Used as intended

__ Used less than intended

__ Pushed plunger more slowly than usual

__ Pushed plunger partway and waited

__ Sniffed/tooted instead of injecting

__ Threw away drug

__ Sold the drug

__ Shared test results with other people

__ Don't Know

__ Refuse to Answer

__ Not Applicable

B148. **Does using a fentanyl test strip make you feel better able to protect yourself from overdose?**

1 Yes

0 No

7 Don't Know

8 Refuse to Answer

9 Not Applicable

**C. Syringe Access and Injection Practices**

**Now I'm going to ask you some questions about your experiences with syringe access.**

C1. **Where did you get most of the syringes you used for injecting drugs in the last 3 months?** (Choose one)

Needle exchange program 01

Needle distribution program or outreach worker 02

From someone who goes to the needle exchange 03

Bought from a pharmacy 04

Bought or got from an unauthorized sources (street, a friend) 05

My own prescription for syringes 06

Shooting gallery or drug dealer 07

Other 08

Don't Know 77

Refuse to Answer 88

If C1 is not equal to 8, then skip to C3.

C2. **Please specify the other place where you obtained most of your needles in the last three months.**

|__|__|__|__|__|__|__|__|__|__|__|__|__|__|__|__|__|__|__|__|__|__|__|__|__|__|__|__|__|__|__|__|__|__|__|__|__|__|__|__|

C3. **In the last 3 months, how many new needles have you obtained for your own use?**

__ __ __ __ __

99997 Don't Know

99998 Refuse to Answer

99999 Not Applicable

C4. **On average, when you get a brand new, never-used needle, how many times do you inject with it before you stop using it or get rid of it?**

|__|__|__|

Don't Know 777

Refuse to Answer 888

**The next several questions are about behaviors related to injection drug use.**

C5. **In the last 3 months**, **how many** **times** **did you give or loan syringes/needles that you had used to someone else (including a close friend or lover) who then used them?**

Times |__|__|__|__|

Don't Know 7777

Refuse to Answer 8888

C6. **In the last 3 months**, **how many** **times** **did you inject using syringes/needles that** **you know** **had been used by someone else (including a close friend or lover)?**

Times |__|__|__|__|

Don't Know 77777

Refuse to Answer 88888

Not Applicable 99999

C7. **In the last 3 months, have you shared a cooker, spoon, or shaker that someone else used before you?**

Yes 1

No 0

Don't Know 7

Refuse to Answer 8

C8. **In the last 3 months, have you used rinse or mix water that someone else used before you?**

Yes 1

No 0

Don't Know 7

Refuse to Answer 8

C9. **In the last 3 months, have you used a filter or cotton that someone else used before you?**

Yes 1

No 0

Don't Know 7

Refuse to Answer 8

If C9 is equal to 0, then skip to C11.

C10. **In the last 3 months, how frequently have you used a cotton or filter that had been used by someone else?** (Choose one)

0 Less than once a month

1 1 to 4 times a month

2 2 to 6 times a week

3 Once a day

4 2 or more times a day

7 Don't Know

8 Refuse to Answer

9 Not Applicable

C11. **In the last 3 months, have you re-used your own cotton or filter? That is, saved it and then re-wetted it to get residual drug from it or just used it again because an unused cotton or filter was unavailable.**

1 Yes

0 No

7 Don't Know

8 Refuse to Answer

9 Not Applicable

If C11 is equal to 0, then skip to C13.

C12. **In the last 3 months, how frequently have you re-used a cotton or filter?** (Choose one)

0 Less than once a month

1 1 to 4 times a month

2 2 to 6 times a week

3 Once a day

4 2 or more times a day

7 Don't Know

8 Refuse to Answer

9 Not Applicable

C13. **In the last 3 months, have you licked the needle or wetted or moisten a needle with saliva/spit prior to injecting with a needle?**

1 Yes

0 No

7 Don't Know

8 Refuse to Answer

9 Not Applicable

If C13 is equal to 0, then skip to C15.

C14. **In the last 3 months, how often did you wet or moisten a needle with saliva/spit before injecting yourself?** (Choose one)

0 None

1 Some

2 About half

3 Most

4 All

7 Don't Know

8 Refuse to Answer

9 Not Applicable

C15. **In the last 3 months, have you used your saliva/spit to wipe or "clean" a spot on your body that you then injected into?** (Did you clean a place to inject on your body with saliva or spit prior to injecting into that spot?)

1 Yes

0 No

7 Don't Know

8 Refuse to Answer

9 Not Applicable

If C15 is equal to 0, then skip to C17.

C16. **In the last 3 months, how often did you wipe your injection site with saliva/spit prior to injecting?** (Choose one)

0 None

1 Some

2 About half

3 Most

4 All

7 Don't Know

8 Refuse to Answer

9 Not Applicable

C17. **In the last 3 months, did you inject another person?** (**Count only injection of illicit substances or misuse of licit substances**)

Yes 1

No 0

Don't Know 7

Refuse to Answer 8

Not Applicable 9

C18. **In the last 3 months, have you been injected by another person?** (**Count only injection of illicit substances or misuse of licit substances.**)

1 Yes

0 No

7 Don't Know

8 Refuse to Answer

9 Not Applicable

C19. **In the last 3 months, how often do you inject in public places (e.g., a park, alley, parking lot) or other place where other people can see you inject?** (**Injecting alone in your tent would not be considered public injection.**) (Choose one)

0 Never (0% of the time)

1 Occasionally (less than 25% of the time)

2 Sometimes (25% to 74% of the time)

3 Usually (75% to 99% of the time)

4 Always (100% of the time)

7 Don't Know

8 Refuse to Answer

9 Not Applicable

C20. **In the last 3 months, how many times have you rushed your injection because you were concerned someone would see or interrupt you?**

__ __ __ __

9997 Don't Know

9998 Refuse to Answer

9999 Not Applicable

C21. **In the last 3 months, in which of the following places have you injected?** (Check all that apply)

__ Your own house/apartment

__ A friend house/apartment

__ An abandoned building

__ A car or other vehicle

__ A shooting gallery

__ A hotel or motel room

__ In public - street, park or alley

__ In a tent

__ In a public restroom

__ In a safe injection facility, safe consumption site

__ In a refresh center (restroom & showers)

__ In a homeless shelter

__ Don't Know

__ Refuse to Answer

__ Not Applicable

C22. **In the last 3 months, where have you usually been located when you injected?** (Choose one)

00 Your own house/apartment

01 A friend's house/apartment

02 An abandoned building

03 A car or other vehicle

04 A shooting gallery

05 A hotel or motel room

06 In public - street, park or alley

07 In a tent

08 In a public restroom

09 In a safe injection facility/safe consumption site

10 In a refresh center (restroom & showers)

11 In a homeless shelter

97 Don't Know

98 Refuse to Answer

99 Not Applicable

**For purposes of the next questions, we have defined supervised consumption site as a place where people come to use their own drugs under the supervision of trained workers. People can use in this place, have access to sterile equipment (syringes, cotton, cookers) and receive referrals to health or social services.**

C23. **Have you ever used drugs in a supervised consumption site?** 1 Yes

0 No

7 Don't Know

8 Refuse to Answer

9 Not Applicable

If C23 is equal to 0, then skip to instruction before D1.

C24. **In what year did you first use a supervised consumption site (or safe injection or drug use facility)?**

__ __ __ __ yyyy

2097 Don't Know (Year)

2098 Refuse to Answer (Year)

2099 Not Applicable (Year)

C25. **In what city was this supervised consumption room located?**

__ __ __ __ __ __ __ __ __ __ __ __ __ __ __ __ __ __ __ __ __ __ __ __ __ __ __ __ __ __ __ __ __ __ __ __ __ __ __ __

C26. **In the last 3 months, have you used drugs in a supervised consumption site?**

1 Yes

0 No

7 Don't Know

8 Refuse to Answer

9 Not Applicable

If C26 is equal to 0, then skip to instruction before D1.

C27. **In the last 3 months, how many times have you used drugs at a supervised consumption site?**

__ __ __

997 Don't Know

998 Refuse to Answer

999 Not Applicable

If C27 is equal to 0, then skip to instruction before D1.

C28. **In the last 3 months, how many times have you injected drugs at a supervised consumption site?**

__ __

97 Don't Know

98 Refuse to Answer

99 Not Applicable

C29. **In the last 3 months, how many times have you smoked drugs at a supervised consumption site?**

__ __

97 Don't Know

98 Refuse to Answer

99 Not Applicable

C30. **In the last 3 months, how many times have you snorted or swallowed drugs at a supervised consumption site?**

__ __

97 Don't Know

98 Refuse to Answer

99 Not Applicable

**D. OVERDOSE**

**The following items are about overdoses you have experienced or witnessed.**

D1. **In the last 3 months, have you overdosed?** 1 Yes

0 No

7 Don't Know

8 Refuse to Answer

9 Not Applicable

If D1 is equal to 0, then skip to D6.

D2. **In the last 3 months, how many times did you overdose?** __ __

97 Don't Know

98 Refuse to Answer

99 Not Applicable

D3. **What drugs had you been using prior to your last overdose(s)? (Include substances and alcohol that you had used in the prior 4 hours before the overdose)** (Check all that apply)

__ Crack cocaine

__ Powder cocaine

__ Heroin

__ Fentanyl

__ Prescription opioids

__ Methamphetamine

__ Speedball

__ Goofball

__ Prescription stimulant

__ Prescription tranquilizer/Benzodiazepines

__ Cannabis

__ Methadone

__ Buprenorphine

__ Alcohol

__ Prescription sedative/barbiturate

__ Other drug

__ Don't Know

__ Refuse to Answer

__ Not Applicable

If D3P is equal to 0, then skip to D5.

D4. **What was this other drug that you used prior to overdosing in the last 3 months?**

__ __ __ __ __ __ __ __ __ __ __ __ __ __ __ __ __ __ __ __ __ __ __ __ __ __ __ __ __ __ __ __ __ __ __ __ __ __ __ __

D5. **When you overdosed in the last 3 months, did any of the following occur in response to your overdose?** (Check all that apply)

__ Received naloxone

__ 911 was called

__ Transported to ER

__ Received rescue breathing/CPR

__ Was examined by EMS

__ Was offered MOUD TX

__ Contact with police

__ Arrested

__ Don't Know

__ Refuse to Answer

__ Not Applicable

D6. **In the last 3 months, have you witnessed a heroin, fentanyl, or prescription opioid overdose?**

1 Yes

0 No

7 Don't Know

8 Refuse to Answer

9 Not Applicable

If D6 is equal to 0, then skip to instruction before E1.

D7. **In the last 3 months, how many heroin or opioid overdoses have you witnessed?**

__ __ __ __ Overdoses

9997 Don't Know

9998 Refuse to Answer

9999 Not Applicable

D8. **In the last 3 months, how many of these witnessed overdoses did you or someone else who was not an EMT or police officer use naloxone or narcan to reverse the overdose?**

__ __ __ __ Times

9997 Don't Know

9998 Refuse to Answer

9999 Not Applicable

If D8 is equal to 0, then skip to D10.

D9. **Of the overdoses you witnessed where naloxone was used, did any of them require more than one dose to revive the person to the best of your knowledge?**

1 Yes

0 No

7 Don't Know

8 Refuse to Answer

9 Not Applicable

D10. **In the last 3 months, how many of these witnessed overdoses did you or someone else call 911?**

__ __ __ __

9997 Don't Know

9998 Refuse to Answer

9999 Not Applicable

D11. **In the last 3 months, how many of these witnessed overdoses resulted in the person who overdosed being transported to the emergency room?**

__ __ __ __

9997 Don't Know

9998 Refuse to Answer

9999 Not Applicable

**E. WITHDRAWAL**

**The following questions are about withdrawal symptoms related to substance use.**

E1. **In the last 3 months, have you experienced restlessness, bone or muscle aches, runny nose, sweating, cold or hot flashes, anxiety, teary eyes, stomach cramps, nausea, diarrhea, vomiting or other symptoms due to withdrawal from heroin or opiates?** (Also, referred to as being "dopesick.")

1 Yes

0 No

7 Don't Know

8 Refuse to Answer

9 Not Applicable

If E1 is equal to 0, then skip to instruction before E4.

E2. **In the last 3 months, how many times have you experienced heroin or opiate withdrawal or withdrawal symptoms?**

__ __ __ __ Times

9997 Don't Know

9998 Refuse to Answer

9999 Not Applicable

E3. **In the last 3 months, when you were in heroin or opiate withdrawal, how painful were your symptoms?** (Choose one)

0 Not painful

1 Somewhat painful

2 Moderately painful

3 Very painful

4 Extremely painful

7 Don't Know

8 Refuse to Answer

9 Not Applicable

If METSUM is equal to 0, then skip to instruction before E7.

E4. **In the last 3 months, have you experienced trouble sleeping, concentrating, being tired, irritable, agitated, anxious, sad, depressed, or unable to do things you normally do because of withdrawal from amphetamines, methamphetamines, or speed?**

1 Yes

0 No

7 Don't Know

8 Refuse to Answer

9 Not Applicable

If E4 is equal to 0, then skip to instruction before E7.

E5. **In the last 3 months, how many times have you experienced withdrawal or withdrawal symptoms due to amphetamine, methamphetamine or speed?**

__ __ __ __ Times

9997 Don't Know

9998 Refuse to Answer

9999 Not Applicable

E6. **In the last 3 months, when you were in amphetamine, methamphetamine or speed withdrawal, how painful were your symptoms?** (Choose one)

0 Not painful

1 Somewhat painful

2 Moderately painful

3 Very painful

4 Extremely painful

7 Don't Know

8 Refuse to Answer

9 Not Applicable

If COKSUM is equal to 0, then skip to instruction before F1.

E7. **In the last 3 months, have you experienced trouble sleeping or sleeping too much, not being able to concentrate, being irritable, tense, sad, unable to enjoy things you normally do or eating too little or too much due to withdrawal from cocaine or crack cocaine?**

1 Yes

0 No

7 Don't Know

8 Refuse to Answer

9 Not Applicable

If E7 is equal to 0, then skip to instruction before F1.

E8. **In the last 3 months, how many times have you experienced withdrawal or withdrawal symptoms due to cocaine or crack cocaine?**

__ __ __ __ Times

9997 Don't Know

9998 Refuse to Answer

9999 Not Applicable

E9. **In the last 3 months, when you were in powdered cocaine or crack cocaine withdrawal, how painful were your symptoms?** (Choose one)

0 Not painful

1 Somewhat painful

2 Moderately painful

3 Very painful

4 Extremely painful

7 Don't Know

8 Refuse to Answer

9 Not Applicable

**F. SEXUAL BEHAVIOR**

**Now I would like to ask you some personal questions about your sexual behavior. We realize that this is a very personal subject, but your answers are very important to our research. Your answers will remain completely confidential, and I remind you that your name isn't written anywhere on this questionnaire. You may refuse to answer any question you wish, but it is important that you be as honest as you can.

In the following questions, by "having sex" we mean having vaginal or anal sex (or insertive/receptive sex). We are not interested in sexual activities that involved only oral sex, petting, mutual masturbation, or using sex toys such as dildos.**

F1. **Have you had any sex partners in the last 3 months?**

Yes 1

No 0

Don't Know 7

Refuse to Answer 8

Not Applicable 9

If F1 is equal to 0, then skip to F18.

F2. **How many sex partners did you have in the last 3 months?** __ __ __ __

9997 Don't Know

9998 Refuse to Answer

9999 Not Applicable

F3. **In the last 3 months, did you have a steady sex partner?**

Yes 1

No 0

Don't Know 7

Refuse to Answer 8

Not Applicable 9

If F3 is equal to 0, then skip to F8.

F4. **In the last 3 months, when you had sex with your steady sex partner(s), how often were you under the influence of drugs or alcohol?** (Choose one)

0 Never (0% of the time)

1 Occasionally (less than 25% of the time)

2 Sometimes (25% to 74% of the time)

3 Usually (75% to 99% of the time)

4 Always (100% of the time)

7 Don't Know

8 Refuse to Answer

9 Not Applicable

F5. **How frequently did you use a condom when having vaginal or anal sex with your steady sex partner?** (Choose one)

Never (0% of the time) 0

Occasionally (less than 25% of the time) 1

Sometimes (25% to 74% of the time) 2

Usually (75% to 99% of the time) 3

Always (100% of the time) 4

Don't Know 7

Refuse to Answer 8

Not Applicable 9

F6. **Does your steady sex partner use drugs?** 1 Yes

0 No

7 Don't Know

8 Refuse to Answer

9 Not Applicable

If F6 is equal to 0, then skip to F8.

F7. **Does your steady sexual partner inject drugs?**

Yes 1

No 0

Don't Know 7

Refuse to Answer 8

Not Applicable 9

F8. **In the last 3 months, did you have any sex partners who paid you in cash or drugs for sex?**

Yes 1

No 0

Don't Know 7

Refuse to Answer 8

Not Applicable 9

If F8 is equal to 0, then skip to F13.

F9. **In the last 3 months, when you had sex with your paying sex partner(s), how often were you under the influence of drugs or alcohol?** (Choose one)

0 Never (0% of the time)

1 Occasionally (less than 25% of the time)

2 Sometimes (25% to 74% of the time)

3 Usually (75% to 99% of the time)

4 Always (100% of the time)

7 Don't Know

8 Refuse to Answer

9 Not Applicable

F10. **How frequently did you use condoms when having vaginal or anal sex with your paying sex partners?** (Choose one)

Never (0% of the time) 0

Occasionally (less than 25% of the time) 1

Sometimes (25% to 74% of the time) 2

Usually (75% to 99% of the time) 3

Always (100% of the time) 4

Don't Know 7

Refuse to Answer 8

Not Applicable 9

F11. **Did any of your paying sex partners use drugs?** 1 Yes

0 No

7 Don't Know

8 Refuse to Answer

9 Not Applicable

If F11 is equal to 0, then skip to F13.

F12. **Did any of your paying sex partner(s) inject drugs?**

Yes 1

No 0

Don't Know 7

Refuse to Answer 8

Not Applicable 9

F13. **In the last 3 months, did you have a casual sex partner (a sex partner who was not steady and not paid)?**

Yes 1

No 0

Don't Know 7

Refuse to Answer 8

Not Applicable 9

If F13 is equal to 0, then skip to F18.

F14. **In the last 3 months, when you had sex with your casual sex partner(s), how often were you under the influence of drugs or alcohol?** (Choose one)

0 Never (0% of the time)

1 Occasionally (less than 25% of the time)

2 Sometimes (25% to 74% of the time)

3 Usually (75% to 99% of the time)

4 Always (100% of the time)

7 Don't Know

8 Refuse to Answer

9 Not Applicable

F15. **How frequently did you use a condom when having vaginal or anal sex with your casual sex partner(s)?** (Choose one)

Never (0% of the time) 0

Occasionally (less than 25% of the time) 1

Sometimes (25% to 74% of the time) 2

Usually (75% to 99% of the time) 3

Always (100% of the time) 4

Don't Know 7

Refuse to Answer 8

Not Applicable 9

F16. **Did any of your casual sex partners use drugs?** 1 Yes

0 No

7 Don't Know

8 Refuse to Answer

9 Not Applicable

If F16 is equal to 0, then skip to F18.

F17. **Did any of your casual sexual partner(s) inject drugs?**

Yes 1

No 0

Don't Know 7

Refuse to Answer 8

Not Applicable 9

F18. **What do you consider your sexual orientation to be?***(Read list aloud.)* (Choose one)

Heterosexual 1

Gay or lesbian 2

Bisexual 3

Asexual - nonsexual 4

Don't Know 7

Refuse to Answer 8

Not Applicable 9

**G. HEALTH**

**The next set of questions are about your health, use of preventive services, and attitudes toward treatments,**

G19. **Have you ever received a vaccination to protect you from Hepatitis A virus?**

1 Yes

0 No

7 Don't Know

8 Refuse to Answer

9 Not Applicable

G20. **Has a doctor, nurse, or health provider ever told you that you were infected with hepatitis A virus?**

1 Yes

0 No

7 Don't Know

8 Refuse to Answer

9 Not Applicable

G21. **Has a doctor, nurse, or counselor ever told you that you are HIV positive?**

Yes 1

No 0

Don't Know 7

Refuse to Answer 8

If G21 is equal to 0, then skip to instruction before G23.

G22. **Are you currently receiving treatment for HIV by taking ART medications and visiting your physician once every 6 months?**

1 Yes

0 No Skip to instruction before G24

7 Don't Know

8 Refuse to Answer

9 Not Applicable Skip to instruction before G24

If G21 is equal to 1, then skip to G34.

If G22 is equal to 0, then skip to G34.

G23. **Have you ever heard of a pill that is safe and effective at lowering transmission of HIV if taken daily prior to exposure to HIV (also known as PrEP or pre-exposure prophylaxis)?**

1 Yes

0 No

7 Don't Know

8 Refuse to Answer

9 Not Applicable

If G23 is equal to 0, then skip to G25.

G24. **Are you currently taking a daily pill to lower your risk for HIV transmission? This pill is typically referred to as PrEP or pre-exposure prophylaxis.**

1 Yes

0 No

7 Don't Know

8 Refuse to Answer

9 Not Applicable

If G24 is equal to 1, then skip to G26.

G25. **Would you be willing to take a once a day pill, everyday to lower your risk of becoming HIV infected by 90%?**

1 Yes

0 No

7 Don't Know

8 Refuse to Answer

9 Not Applicable

G26. **Have you ever tried to get PrEP?** 1 Yes

0 No

7 Don't Know

8 Refuse to Answer

9 Not Applicable

If G26 is equal to 0, then skip to G31.

G27. **Did you ever receive a prescription for PrEP?** 1 Yes

0 No

7 Don't Know

8 Refuse to Answer

9 Not Applicable

If G27 is equal to 0, then skip to G29.

G28. **Were you able to fill any PrEP prescription that you have received?** 1 Yes

0 No

7 Don't Know

8 Refuse to Answer

9 Not Applicable

If G28 is equal to 0, then skip to G30.

G29. **Did you ever take the PrEP prescribed to you?** 1 Yes

0 No

7 Don't Know

8 Refuse to Answer

9 Not Applicable

G30. **Have you ever taken PrEP not prescribed to you?** 1 Yes

0 No

7 Don't Know

8 Refuse to Answer

9 Not Applicable

G31. **Have you ever heard of a pill that can be taken for 30 days after a single high-risk event to stop HIV seroconversion (also call PEP or post-exposure prophylaxis)?**

1 Yes

0 No

7 Don't Know

8 Refuse to Answer

9 Not Applicable

If G31 is equal to 0, then skip to G33.

G32. **In the last 3 months, have you ever taken an antiretroviral pill after a single high-risk event to stop HIV seroconversion?**

1 Yes

0 No

7 Don't Know

8 Refuse to Answer

9 Not Applicable

If G32 is equal to 1, then skip to G34.

G33. **Would you be willing to take a once a day pill for 30 days, after a single high-risk event to prevent HIV seroconversion?**

1 Yes

0 No

7 Don't Know

8 Refuse to Answer

9 Not Applicable

G34. **Have you ever tested positive for hepatitis C virus or HCV infection or been told by a health care professional that you are infected with the hepatitis C virus or HCV?**

1 Yes

0 No

7 Don't Know

8 Refuse to Answer

9 Not Applicable

If G34 is equal to 0, then skip to G36.

G35. **Have you ever or are you currently receiving treatment for hepatitis C virus or HCV infection?**

1 Yes

0 No

7 Don't Know

8 Refuse to Answer

9 Not Applicable

G36. **Have you ever been diagnosed with mental health disorder or a psychiatric illness (e.g. major depression, bipolar disorder, anxiety, PTSD, schizophrenia)?**

1 Yes

0 No

7 Don't Know

8 Refuse to Answer

9 Not Applicable

If G36 is equal to 0, then skip to G42.

G37. **Which of the following mental health diagnoses have you received? (Read list)** (Check all that apply)

__ Depression

__ Bipolar disorder/manic depression

__ Schizophrenia

__ PTSD or Post traumatic stress disorder

__ Anxiety

__ Other psychiatric illness

__ Don't Know

__ Refuse to Answer

__ Not Applicable

If G37F is equal to 0, then skip to G39.

G38. **What was this other mental health disorder or psychiatric illness?**

__ __ __ __ __ __ __ __ __ __ __ __ __ __ __ __ __ __ __ __ __ __ __ __ __ __ __ __ __ __ __ __ __ __ __ __ __ __ __ __ __ __ __ __ __ __ __ __ __ __ __ __ __ __ __ __ __ __ __ __ __ __ __ __ __ __ __ __ __ __ __ __ __ __ __ __ __ __ __ __ __ __ __ __ __ __ __ __ __ __ __ __ __ __ __ __ __ __ __ __

G39. **Are you currently taking any doctor prescribed medications for any of your mental health diagnoses?**

1 Yes

0 No

7 Don't Know

8 Refuse to Answer

9 Not Applicable

If G39 is equal to 0, then skip to G42.

G40. **For which diagnoses are you prescribed medications?** (Check all that apply)

__ Depression

__ Bipolar disorder

__ Schizophrenia

__ PTSD

__ Anxiety

__ Other

__ Don't Know

__ Refuse to Answer

__ Not Applicable

If G40F is equal to 0, then skip to G42.

G41. **What is the other diagnosis that you have been prescribed medications for?**

__ __ __ __ __ __ __ __ __ __ __ __ __ __ __ __ __ __ __ __ __ __ __ __ __ __ __ __ __ __ __ __ __ __ __ __ __ __ __ __ __ __ __ __ __ __ __ __ __ __

G42. **In the last 3 months, have you been detained under a 5150 order or involuntarily hospitalized due to a mental health problem?**

1 Yes

0 No

7 Don't Know

8 Refuse to Answer

9 Not Applicable

If G42 is equal to 0, then skip to G44.

G43. **How many times were you detained in the last 3 months?** __ __

97 Don't Know

98 Refuse to Answer

99 Not Applicable

G44. **In the last 3 months, did you voluntarily hospitalize yourself due to a mental health disorder?**

1 Yes

0 No

7 Don't Know

8 Refuse to Answer

9 Not Applicable

G45. **In the last 3 months, have you had an abscess related to injection drug use, even if it did not "come to a head," drain, or require treatment of any kind?**

1 Yes

0 No

7 Don't Know

8 Refuse to Answer

9 Not Applicable

If G45 is equal to 0, then skip to G47.

G46. **How many abscesses have you had in the last 3 months?**  (It is fine to estimate.)

__ __ __ __

9997 Don't Know

9998 Refuse to Answer

9999 Not Applicable

G47. **In the past 3 months, have you experienced high fevers, shaking chills, night sweats or other symptoms due to a potentially untreated infection?**

1 Yes

0 No

7 Don't Know

8 Refuse to Answer

9 Not Applicable

If G47 is equal to 0, then skip to G49.

G48. **Were those symptoms accompanied by increased shortness of breath, weight loss, back pain, chest pain, bloody urine, painful spots on your fingers or toes, painless red spots on your fingers or toes, swelling in your legs, loss of or changes in vision, or new headaches?**

1 Yes

0 No

7 Don't Know

8 Refuse to Answer

9 Not Applicable

G49. **Have you ever been diagnosed with infective endocarditis (an infection to your heart valve)?**

1 Yes

0 No

7 Don't Know

8 Refuse to Answer

9 Not Applicable

If G49 is equal to 0, then skip to G54.

G50. **Did you complete the full treatment course prescribed to you? Usually, this includes 4-8 weeks of antibiotics and sometimes surgery to replace a heart valve.**

1 Yes

0 No

7 Don't Know

8 Refuse to Answer

9 Not Applicable

If G50 is equal to 0, then skip to instruction before G52.

G51. **Why did you not complete the full treatment course?** (Check all that apply)

__ Could not pay for treatment

__ Did not feel treatment was appropriate

__ Did not receive treatment for withdrawal symptoms

__ Did not think treatment was necessary

__ Was not treated fairly by medical staff

__ Was kicked out of care for any reason

__ Was kicked out of care for drug use

__ Other

__ Don't Know

__ Refuse to Answer

__ Not Applicable

If G51H is equal to 0, then skip to G53.

G52. **For what other reasons did you not complete treatment?**

__ __ __ __ __ __ __ __ __ __ __ __ __ __ __ __ __ __ __ __

G53. **In the last 3 months, has a doctor, nurse, or medical provider told you that you have infective endocarditis (an infection in your heart valve)?**

1 Yes

0 No

7 Don't Know

8 Refuse to Answer

9 Not Applicable

G54. **Has a health care professional ever told you that you have [Diabetes, High blood pressure, ...]?**

Yes 1

No 0

Don't Know 7

Refuse to Answer 8

Not Applicable 9

|  | Diabetes | High blood pressure/hypertension | High cholesterol | Congestive Heart Failure/Heart Attack | Stroke | MRSA |
| --- | --- | --- | --- | --- | --- | --- |
| G54 | \|__\| | \|__\| | \|__\| | \|__\| | \|__\| | \|__\| |

G55. **In the last 3 months, how many times have you been seen in an emergency room for a health problem?**

__ __

97 Don't Know

98 Refuse to Answer

99 Not Applicable

G56. **How many times have you been hospitalized overnight for any health issue in the last 3 months?**

__ __ __ __ Times

9997 Don't Know

9998 Refuse to Answer

9999 Not Applicable

If G56 is equal to 0, then skip to instruction before G58.

G57. **How many nights did you spend in the hospital in the last 3 months?**

__ __ __

997 Don't Know

998 Refuse to Answer

999 Not Applicable

If A3 is equal to 0, then skip to instruction before G59&C.

If A3 is greater than 1, then skip to instruction before G59&C.

G58. **In the last 3 months, have you experienced any of the following gynecological symptoms or conditions? (Read list - selected not applicable if none are mentioned)** (Check all that apply)

__ Need for a pregnancy test

__ Concern about a pregnancy

__ Miscarriage

__ Missed a period for a month or more

__ Need for an abortion

__ An abortion

__ Don't Know

__ Refuse to Answer

__ Not Applicable

The next items about your experiences during the first 12 months of the COVID-19 pandemic.

G59. **At any point during [Apr to June 2020, Jul to Sep 2020, ...] did you receive a COVID-19 test?**

Yes 1

No 0

Don't Know 7

Refuse to Answer 8

Not Applicable 9

G60. **At any point during [Apr to June 2020, Jul to Sep 2020, ...] did you test positive for COVID-19?**

Yes 1

No 0

Don't Know 7

Refuse to Answer 8

Not Applicable 9

G61. **At any point during [Apr to June 2020, Jul to Sep 2020, ...] did you have to quarantine because of potential exposure to someone who was COVID-19?**

Yes 1

No 0

Don't Know 7

Refuse to Answer 8

Not Applicable 9

G62. **At any point during [Apr to June 2020, Jul to Sep 2020, ...] were you incarcerated in jail or prison?**

Yes 1

No 0

Don't Know 7

Refuse to Answer 8

Not Applicable 9

G63. **At any point during [Apr to June 2020, Jul to Sep 2020, ...] were you housed to prevent exposure to COVID-19? Probe: Housed in project roomkey or other temporary housing to help you shelter-in-place?**

Yes 1

No 0

Don't Know 7

Refuse to Answer 8

Not Applicable 9

|  | Apr to June 2020 | Jul to Sep 2020 | Oct to Dec 2020 | Jan to Mar 2021 |
| --- | --- | --- | --- | --- |
| G59 | \|__\| | \|__\| | \|__\| | \|__\| |
| G60 | \|__\| | \|__\| | \|__\| | \|__\| |
| G61 | \|__\| | \|__\| | \|__\| | \|__\| |
| G62 | \|__\| | \|__\| | \|__\| | \|__\| |
| G63 | \|__\| | \|__\| | \|__\| | \|__\| |

G64. **Have you received a vaccination for COVID-19?** 1 Yes

0 No

7 Don't Know

8 Refuse to Answer

9 Not Applicable

If G64 is equal to 0, then skip to G66.

G65. **What month and year did you receive the COVID-19 vaccine?**

__ __ __ __ __ __ __ __ __ __ __ __ __ __ __ __ __ __ __ __

G66. **Do you have any of the following types of health benefits? (Read List)** (Check all that apply)

Private insurance |__|

Medicaid or Medi-Cal |__|

Medicare |__|

Veterans benefits |__|

Covered California |__|

Other |__|

No insurance |__|

Don't Know |__|

Refuse to Answer |__|

Not Applicable |__|

If G66F is equal to 0, then skip to instruction before G68.

G67. **What is this other kind of health insurance?**

__ __ __ __ __ __ __ __ __ __ __ __ __ __ __ __ __ __ __ __ __ __ __ __ __ __ __ __ __ __ __ __ __ __ __ __ __ __ __ __ __ __ __ __ __ __ __ __ __ __

**The next half dozen questions are about chronic pain.**

G68. **In the last 3 months, have you experienced any chronic or ongoing pain due to an injury or other ailment (like arthritis or migraine headaches, but not counting withdrawal symptoms)?**

1 Yes

0 No

7 Don't Know

8 Refuse to Answer

9 Not Applicable

If G68 is equal to 0, then skip to instruction before G76.

G69. **How would you rate your chronic physical pain on a 0-9 scale at the present time or right now, where 0 is 'no pain' and 9 is 'pain as bad as it could be?'**

00 No pain

01

02

03

04

05

06

07

08

09 Worst pain

97 Don't Know

98 Refuse to Answer

99 Not Applicable

G70. **In the past 3 months, how intense was your worst chronic physical pain rated on a 0-9 scale, where 0 is 'no pain' and 9 is 'pain as bad as it could be?'**

00 No pain

01

02

03

04

05

06

07

08

09 Worst pain

97 Don't Know

98 Refuse to Answer

99 Not Applicable

G71. **In the past 3 months, on average, how intense was your chronic physical pain rated on a 0-9 scale? (That is your usual pain at times you were experiencing pain.)**

00 No pain

01

02

03

04

05

06

07

08

09 Worst pain

97 Don't Know

98 Refuse to Answer

99 Not Applicable

G72. **About how many DAYS in the last 3 months have you been kept from your usual activities (work, school, housework) because of chronic physical pain?**

__ __ __ DAYS

997 Don't Know

998 Refuse to Answer

999 Not Applicable

G73. **In the past 3 months, how much has this chronic physical pain interfered with your daily activities on a 0-9 scale where 0 is 'no interference' and 9 is 'extreme change'?**

00 No interference

01

02

03

04

05

06

07

08

09 Extreme change

997 Don't Know

998 Refuse to Answer

999 Not Applicable

G74. **In the past 3 months, how much has chronic physical pain changed your ability to take part in recreational, social, and family activities where 0 is 'no change' and 9 is 'extreme change'?**

00 No change

01

02

03

04

05

06

07

08

09 Extreme change

997 Don't Know

998 Refuse to Answer

999 Not Applicable

G75. **In the past 3 months, how has this chronic physical pain changed your ability to work (including housework) where 0 is 'no change' and 9 is 'extreme change'?**

00 No change

01

02

03

04

05

06

07

08

09 Extreme change

997 Don't Know

998 Refuse to Answer

999 Not Applicable

**The next set of questions are about how people have treated you and people like you in the last 3 months.**

G76. **In the last 3 months, how often have people spoken or acted towards you in a way that felt like they were attacking your dignity or demeaning you?** (Choose one)

1 Never

2 1 to 2 times

3 3-10 times

4 About once a month

5 About once or twice a week

6 Daily or more

7 Don't Know

8 Refuse to Answer

9 Not Applicable

If G76 is equal to 1, then skip to G79.

G77. **What aspect of you or your life did you think they were demeaning you about?** (Check all that apply)

__ My drug use

__ The way I use drugs (injection)

__ My personality

__ The way I look

__ My clothing

__ Not having a job

__ Lack of education

__ People I hung out with

__ People I have sex with

__ My race

__ My gender

__ My sexual orientation

__ My weight

__ My personal hygiene (cleanliness)

__ How I earn money

__ Being poor

__ My HIV or HCV status

__ Having children with different partners

__ My housing situation

__ HIV status

__ HCV status

__ Don't Know

__ Refuse to Answer

__ Not Applicable

G78. **Please tell me who are the people and/or groups who have attacked your dignity or demeaned you in the way that hurt you the most?** (Check all that apply)

__ MOTHER

__ FATHER

__ SIBLING

__ EX-SPOUSE/LOVER

__ OTHER RELATIVES

__ FRIENDS

__ NEIGHBORS

__ BOSS/EMPLOYER

__ CO-WORKERS

__ DRUG DEALER

__ COUNSELOR/SOCIAL WORKER

__ PROBATION/PAROLE OFFICER

__ POLICE OFFICER

__ STOREOWNER

__ STRANGER

__ HOSPITAL OR CLINIC STAFF/DOCTOR

__ DRUG TREATMENT STAFF

__ SECURITY GUARD

__ PHARMACY STAFF

__ SEP/SSP STAFF

__ Don't Know

__ Refuse to Answer

__ Not Applicable

G79. **In the last 3 months, how often have you been present when someone spoke or acted towards someone else in a way that attacked their dignity or demeaned them?** (Choose one)

1 Never

2 1 to 2 times

3 3-10 times

4 About once a month

5 About once or twice a week

6 Daily or more

7 Don't Know

8 Refuse to Answer

9 Not Applicable

If G79 is equal to 1, then skip to instruction before H1.

G80. **What aspect of their life did you think they were being demeaned about?** (Check all that apply)

__ Their drug use

__ The way they use drugs (injection)

__ Their personality

__ The way they look

__ Their clothing

__ Not having a job

__ Lack of education

__ People they hung out with

__ People they have sex with

__ Their race

__ Their gender

__ Their sexual orientation

__ Their weight

__ Their personal hygiene (cleanliness)

__ How they earn money

__ Being poor

__ Their HIV or HCV status

__ Having children with different partners

__ Their housing situation

__ Their HIV status

__ Their HCV status

__ Don't Know

__ Refuse to Answer

__ Not Applicable

G81. **Please tell me who are the people and/or groups who have attacked this other person's dignity or demeaned them in the way that hurt them the most?** (Check all that apply)

__ MOTHER

__ FATHER

__ SIBLING

__ EX-SPOUSE/LOVER

__ OTHER RELATIVES

__ FRIENDS

__ NEIGHBORS

__ BOSS/EMPLOYER

__ CO-WORKERS

__ DRUG DEALER

__ COUNSELOR/SOCIAL WORKER

__ PROBATION/PAROLE OFFICER

__ POLICE OFFICER

__ STOREOWNER

__ STRANGER

__ HOSPITAL OR CLINIC STAFF/DOCTOR

__ DRUG TREATMENT STAFF

__ SECURITY GUARD

__ PHARMACY STAFF

__ SEP/SSP STAFF

__ Don't Know

__ Refuse to Answer

__ Not Applicable

**H. VIOLENCE**

**The next questions are about threats and violence that may have happened to you in the past 3 months.**

H1. **In the past 3 months, has anybody threatened you with a knife, gun, or other weapon?**

1 Yes

0 No

7 Don't Know

8 Refuse to Answer

9 Not Applicable

H2. **In the past 3 months, has anybody punched, slapped, kicked, or physically hurt you?**

1 Yes

0 No

7 Don't Know

8 Refuse to Answer

9 Not Applicable

H3. **In the past 3 months, has anybody used a knife, gun, club, or other weapon against you?**

1 Yes

0 No

7 Don't Know

8 Refuse to Answer

9 Not Applicable

H4. **In the past 3 months, has somebody used physical force or threats to make you have vaginal, anal, or oral sex with them?**

1 Yes

0 No

7 Don't Know

8 Refuse to Answer

9 Not Applicable

H5. **In the past 3 months, have any of your belongings been stolen?** 1 Yes

0 No

7 Don't Know

8 Refuse to Answer

9 Not Applicable

H6. **In the past 3 months, have you been attacked by a stranger on the street?** 1 Yes

0 No

7 Don't Know

8 Refuse to Answer

9 Not Applicable

**I. Law Enforcement**

**Next I am going to ask you questions about your contacts with security guards, law enforcement, and criminal justice systems.**

I1. **In the last 3 months, have you had any contact with private security guards? (do not count police who work for a city, county or state).**

1 Yes

0 No

7 Don't Know

8 Refuse to Answer

9 Not Applicable

If I1 is equal to 0, then skip to I3.

I2. **How many times have you had contact with security guards in the last 3 months?**

__ __ __ __ Times

9997 Don't Know

9998 Refuse to Answer

9999 Not Applicable

I3. **In the past 3 months, have you had contact with the police?** 1 Yes

0 No

7 Don't Know

8 Refuse to Answer

9 Not Applicable

If I3 is equal to 0, then skip to I8.

I4. **How many times have you had contact with the police in the past 3 months**

__ __ __ __ Times

9997 Don't Know

9998 Refuse to Answer

9999 Not Applicable

If I4 is equal to 0, then skip to I8.

I5. **Have you been arrested in the last 3 months?** 1 Yes

0 No

7 Don't Know

8 Refuse to Answer

9 Not Applicable

If I5 is equal to 0, then skip to I8.

I6. **How many times have you been arrested in the last 3 months?** __ __ Times

97 Don't Know

98 Refuse to Answer

99 Not Applicable

I7. **How many nights have you spent in jail in the last 3 months?** __ __

97 Don't Know

98 Refuse to Answer

99 Not Applicable

I8. **Have you been on probation at any point in the last 3 months?** 1 Yes

0 No

7 Don't Know

8 Refuse to Answer

9 Not Applicable

I9. **Have you been on parole at any point in the last 3 months?** 1 Yes

0 No

7 Don't Know

8 Refuse to Answer

9 Not Applicable

**J. DRUG TREATMENT**

**The next set of questions are about your experiences with substance use treatment.**

J1. **In the last 3 months, have you participated in any treatment program, not counting self-help, NA, CA, or AA?**

1 Yes

0 No

7 Don't Know

8 Refuse to Answer

9 Not Applicable

If J1 is equal to 0, then skip to J5.

J2. **In the last 3 months, have you participated in [Methadone detoxification, Methadone maintenance, ...] ?**

Yes 1

No 0

Refuse to Answer 8

Not Applicable 9

|  | Methadone detoxification | Methadone maintenance | Buprenorphine detox | Buprenorphine maintenance | Outpatient W/O MAT | Inpatient hospital | Residential W/O MAT | Vivitrol/naltrexone |
| --- | --- | --- | --- | --- | --- | --- | --- | --- |
| J2 | \|__\| | \|__\| | \|__\| | \|__\| | \|__\| | \|__\| | \|__\| | \|__\| |

J3. **Are you currently enrolled in any kind of substance use treatment?** 1 Yes

0 No

7 Don't Know

8 Refuse to Answer

9 Not Applicable

J4. **Which treatments are your currently receiving?** (Check all that apply)

__ Methadone detox

__ Methadone maintenance

__ Buprenorphine

__ Outpatient

__ Inpatient hospital

__ Residential

__ Vivitrol/Naltrexone

__ Don't Know

__ Refuse to Answer

__ Not Applicable

J5. **In the last 3 months, have you tried, but been unable to get into drug treatment?**

1 Yes

0 No

7 Don't Know

8 Refuse to Answer

9 Not Applicable

If J5 is equal to 0, then skip to instruction before J7.

J6. **Which types of treatment did you try to enter in the last 3 months?** (Check all that apply)

__ Methadone detoxification

__ Methadone maintenance

__ Buprenorphine (suboxone or subutex) detox

__ Buprenorphine/suboxone maintenance

__ Outpatient

__ Inpatient hospital

__ Residential

__ Vivitrol/naltrexone

__ Don't Know

__ Refuse to Answer

__ Not Applicable

**The following statements refer to heroin, fentanyl, opioids, methamphetamine, cocaine (but not cannabis or alcohol). Please tell me how much you strongly agree, agree, neutral or disagree, or strongly disagree with each statement.**

J7. **My drug use is a problem for me.** (Choose one) 0 Strongly disagree

1 Disagree

2 Neutral

3 Agree

4 Strongly agree

7 Don't Know

8 Refuse to Answer

9 Not Applicable

J8. **My drug use is more trouble than it is worth** (Choose one) 0 Strongly disagree

1 Disagree

2 Neutral

3 Agree

4 Strongly agree

7 Don't Know

8 Refuse to Answer

9 Not Applicable

J9. **My drug use is under control.** (Choose one) 0 Strongly disagree

1 Disagree

2 Neutral

3 Agree

4 Strongly agree

7 Don't Know

8 Refuse to Answer

9 Not Applicable

J10. **I plan to quit using drugs in the next 6 months.** (Choose one) 0 Strongly disagree

1 Disagree

2 Neutral

3 Agree

4 Strongly agree

7 Don't Know

8 Refuse to Answer

9 Not Applicable

J11. **I plan to quit using drugs in the next 30 days.** (Choose one) 0 Strongly disagree

1 Disagree

2 Neutral

3 Agree

4 Strongly agree

7 Don't Know

8 Refuse to Answer

9 Not Applicable

J12. **I am ready to quit using drugs right now.** (Choose one) 0 Strongly disagree

1 Disagree

2 Neutral

3 Agree

4 Strongly agree

7 Don't Know

8 Refuse to Answer

9 Not Applicable

**K. Sleep and subsistence**

**We are almost done.**

K1. **In the last 3 months, how would you rate your sleep quality?** (Choose one) 0 Very bad

1 Fairly bad

2 Fairly good

3 Very good

7 Don't Know

8 Refuse to Answer

9 Not Applicable

K2. **In the last 3 months, how many hours of actual sleep do you usually get per day?**

__ __ HOURS

97 Don't Know (Hours)

98 Refuse to Answer (Hours)

99 Not Applicable (Hours)

K3. **In the last 3 months, has finding shelter been a problem for you usually, sometimes, rarely or never?** (Choose one)

1 Never

2 Rarely

3 Sometimes

4 Usually

7 Don't Know

8 Refuse to Answer

9 Not Applicable

K4. **In the last 3 months, has getting enough to eat been a problem for you usually, sometimes, rarely, or never?** (Choose one)

1 Never

2 Rarely

3 Sometimes

4 Usually

7 Don't Know

8 Refuse to Answer

9 Not Applicable

K5. **In the last 3 months, has obtaining clothes been a problem for you usually, sometimes, rarely, or never?** (Choose one)

1 Never

2 Rarely

3 Sometimes

4 Usually

7 Don't Know

8 Refuse to Answer

9 Not Applicable

K6. **In the last 3 months, has finding a place to wash up been a problem for you usually, sometimes, rarely, or never?** (Choose one)

1 Never

2 Rarely

3 Sometimes

4 Usually

7 Don't Know

8 Refuse to Answer

9 Not Applicable

K7. **In the last 3 months, has finding a place to go to the bathroom been a problem for you usually, sometimes, rarely, or never?** (Choose one)

1 Never

2 Rarely

3 Sometimes

4 Usually

7 Don't Know

8 Refuse to Answer

9 Not Applicable

K8. **FOR INTERVIEWER ONLY; How would you rate the information collected in this interview?** (Choose one)

0 Completely unreliable

1 Not sure if reliable

2 Reliable

7 Don't Know

8 Refuse to Answer

9 Not Applicable

If NUMOPIOID is greater than 0, then skip to end of questionnaire.

**This respondent did not report any injection drug use or no opioid use in the last 3 months. They are ineligible for the study. Thank them for their time and end the interview.**
